# Dispersion matters: Diagnostics and control data computer simulation in Concealed Information Test studies

**DOI:** 10.1371/journal.pone.0240259

**Published:** 2020-10-02

**Authors:** Gáspár Lukács, Eva Specker

**Affiliations:** 1 Department of Cognition, Emotion, and Methods in Psychology, University of Vienna, Vienna, Austria; 2 Department of Security and Crime Science, University College London, London, United Kingdom; University of Zurich, SWITZERLAND

## Abstract

Binary classification has numerous applications. For one, lie detection methods typically aim to classify each tested person either as “liar” or as “truthteller” based on the given test results. To infer practical implications, as well as to compare different methods, it is essential to assess the diagnostic efficiency, such as demonstrating the number of correctly classified persons. However, this is not always straightforward. In Concealed Information Tests (CITs), the key predictor value (*probe-irrelevant difference*) for “truthtellers” is always similar (zero on average), and “liars” are always distinguished by a larger value (i.e., a larger number resulting from the CIT test, as compared to the zero baseline). Thereby, in general, the larger predictor values a given CIT method obtains for “liars” on average, the better this method is assumed to be. This has indeed been assumed in countless studies, and therefore, when comparing the classification efficiencies of two different designs, the mean difference of “liar” predictor values in the two designs were simply compared to each other (hence not collecting “truthteller” data to spare resources). We show, based on the meta-data of 12 different experimental designs collected in response time-based CIT studies, that differences in dispersion (i.e., variance in the data, e.g. the extent of random deviations from the zero average in case of “truthtellers”) can substantially influence classification efficiency–to the point that, in extreme cases, one design may even be superior in classification despite having a larger mean “liar” predictor value. However, we also introduce a computer simulation procedure to estimate classification efficiency in the absence of “truthteller” data, and validate this procedure via a meta-analysis comparing outcomes based on empirical data versus simulated data.

## Introduction

Simplistic polygraph lie detector tests are widely known from reality shows and similar popular media. Though this specific type of testing has been widely documented as flawed [1, 2], there are indeed various scientifically established paradigms to assess truth telling in people [3–5]. All these paradigms use binary classification, where each tested person is typically classified as a liar or a truthteller based on the given test results. To infer practical implications, as well as to compare different methods, it is essential to assess the diagnostic efficiency–as typically measured by area under the receiver operating characteristic curve (AUC) [6, 7]. Properly assessing diagnostic efficiency is a challenge in itself [8–10].

**Fig 1:**
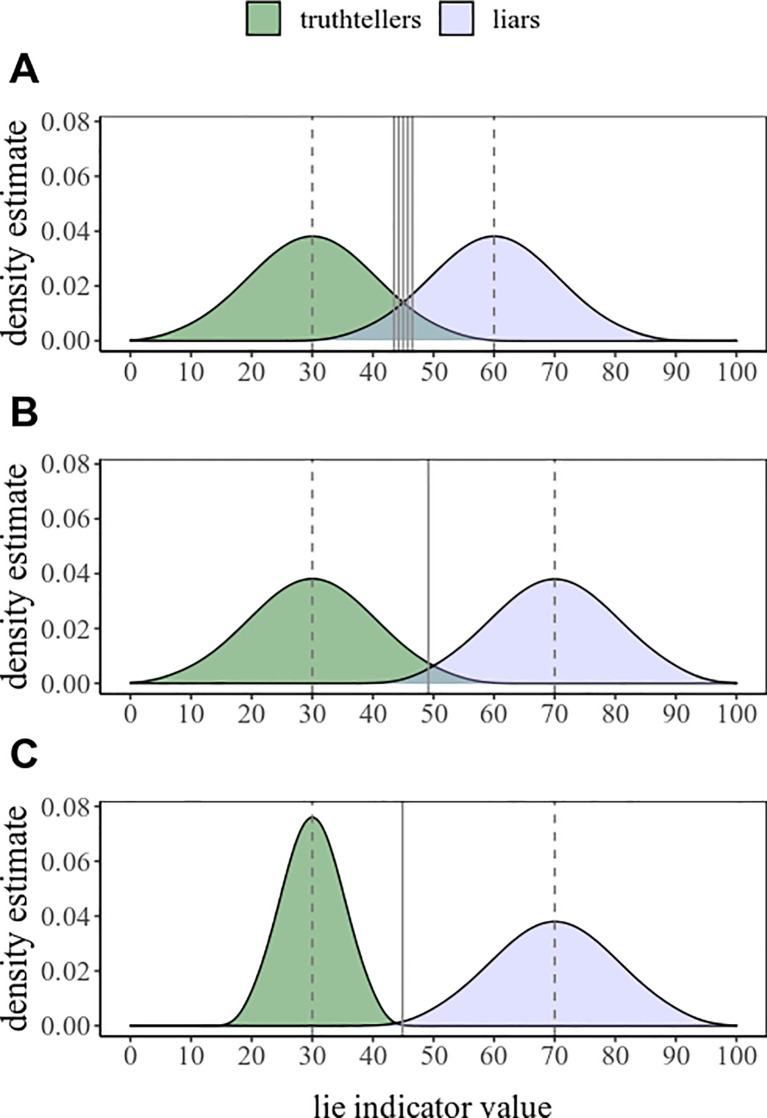
Density plots illustrating dispersions for the obtained lie indicator values. In truthteller and liar conditions, using three different hypothetic lie detection devices: A, B, and C. Note that the decreasing overlap (largest for A, smallest for C) means an increase in efficiency for classification (i.e., the smaller the overlap, the better the classification). Grey solid vertical lines indicate optimal cutoff points for classification. (In panel A, there are multiple equally optimal cutoff points due to the evenly distributed values in the simulation. This is unlikely with empirical data).

For illustration, let us say that we have a hypothetic deception detector device *A*, which, whenever a statement is uttered, displays a “lie indicator value” as a number from zero to a hundred, with larger number indicating larger likelihood of the person having lied (i.e., deception). To empirically assess the diagnostic efficiency of this binary classifier, we recruit 200 people, half telling a lie, and half giving a truthful statement. We find that the liar group indeed receives larger values as compared to the truthful group (average 60 vs. 30, with standard deviations of 10 each, in close to perfect normal distribution). This is illustrated in Fig 1, upper panel A (see S1 Script). Thereby, most liars can be distinguished from truthtellers if we set a particular cutoff value for classification (based on empirical observations, using e.g. Youden’s index [11]): Those who get a value above this number will be classified as liars, and the rest as truthtellers. For example, an optimal cutoff is at value 45, in which case 94 people from each of the two groups are classified correctly, and six from each of the two groups are classified incorrectly.

We could then introduce an improved device *B*, which functions similarly as device *A*, but may provide improved diagnostic ability, that is, higher accuracy in distinguishing liars from truthtellers. We repeat the experiment and find that device *B* is indeed an improvement, providing larger lie indicator values for liars, with an average 70. This is illustrated in Fig 1, panel B–note the decreased overlap between liar and truthteller data. Thereby, the diagnostic efficiency increased even further: At an optimal threshold around 50, altogether only three persons out of the total 200 are misclassified (two truthtellers and one liar in the case of our simulated data). The diagnostic efficiencies of these two devices (*A* and *B*) can then be statistically compared, for example, using DeLong’s test to compare the two AUCs [12, 13]. Similar to simple overall classification accuracy rate–number of correct classifications divided by the number of all classifications–the AUC can range from 0 to 1, where .5 means chance level classification, and 1 means flawless classification–i.e., all guilty and informed innocent classifications can be correctly made based on the given predictor variable, at a given cutoff point. The advantage of the AUC is that it takes into account the distribution of all predictor values, thereby relying less on chance cutoff points and providing more generalizable indication for future samples.

Importantly, the truthteller data is completely identical in these two cases (i.e., identical results are obtained from truthtellers with either device). Now, let us assume that all future experiments using any devices will also result in the same identical truthteller data. In that case, the truthtellers may be omitted to spare resources. To assess potential differences in diagnostic efficiency, the arithmetic means of the two different liar results (using device *A* vs. *B*) can be directly compared using a simple *t*-test (or other, e.g., Bayesian equivalent). If the mean lie indicator value of the liars is larger for device *B*, as compared to device *A*, we may conclude that device *B* has better diagnostic efficiency.

However, we now come to the key point in our paper: Distributional properties must also be taken into account. When the dispersion of the resulting values of either the liar or truthteller group is more narrow (i.e., has smaller data variance; typically reported as standard deviation [SD] to quantify the extent of dispersion [14]), there is less overlap between the two groups, and hence truthtellers and liars can be more efficiently distinguished from each other [9]. For example, we could introduce a device *C*, whose data is identical to that obtained with device *B*, except that the new data for one of the groups is more narrowly distributed, thus the variance of the truthteller data is substantially decreased (SD = 5 instead of 10; Fig 1, panel C). This leads to improved diagnostic ability: At an optimal threshold around 45, all 200 persons are correctly classified; even without any changes in the means of either group.

The aims of the present paper are to: (a) demonstrate that analogous scenarios actually occur in real studies concerning a specific deception detection method, the response time-based Concealed Information Test (RT-CIT [15]), and (b) introduce a simulation approach to create control cases that provides a very close approximation of empirical results and hence can be used to estimate AUCs.

To note, here we focus on RT-CIT, but the same reasoning applies to all CIT paradigms (e.g., polygraph, EEG, etc. [5, 16]). Given the enormous number of studies applying of binary classification (e.g. in medicine [17–19], forecasting [20], or other topics in social sciences [21]), it would be very difficult to spot analogous examples (with constant but variable control data) in different lines of research, but we think it is likely that similar cases do occur. In such cases, the solution presented here may be applied analogously.

### The response time-based Concealed Information Test

The RT-CIT aims to reveal whether a person is concealing knowledge regarding a certain detail [16]. To illustrate the CIT, let us consider a murder case scenario in which the murder weapon is known only to the perpetrator and the investigators. In this case, the CIT could include the actual murder weapon (the probe; e.g., "rifle") and several other weapons (irrelevants; e.g., "knife" and "rope") as the items that would be sequentially presented to a suspect one by one in a random order. When each item has to be responded to with a keypress, the recognition of the probe (in this case, "rifle") by a person who is aware of the relevance of that item will typically result in a slower response to that item than to the irrelevant items [22]. Thereby, based on the probe-irrelevant RT differences, liars who deny recognizing the probe (as a relevant item among the rest) can be distinguished from truthtellers who are truly not familiar with the relevant probe. This difference is conventionally calculated, per each individual test, as the mean of all response times to probes minus the mean of all response times for irrelevants [15, 23].

To note, in deception detection literature, the key conditions in RT-CIT studies are typically labelled “knowledgeable” (here “liar”) and “naive” (here “truthteller”), because in general this method does not assess lying per se, but merely the knowledge of a certain detail. In the literature of diagnostic efficiency assessment or signal detection, a baseline condition (which lacks the material–here: lies–to be detected), such as “truthtellers,” would be called the “negative condition,” or, more often, the control condition, or simply controls, while the opposite (here: “liar”) would be called the “positive condition”, or cases. Throughout the present paper, for consistency and clarity, in context of deception we keep to the illustrative designations “liars” and “truthtellers” that are easy to understand for any reader, and in context of data we write “liar” and “control” data.

While the methodological details of the RT-CIT are not relevant to the present paper, it is crucial that truthtellers (i.e., those not aware of the relevance of the probe) are assumed to see no difference between probe and irrelevant items. Therefore, there should be no probe-irrelevant RT differences, except by accidental variation (i.e., measurement error). Consequently, it has also been found in all studies involving such a condition that each truthteller group has an approximately zero probe-irrelevant RT difference on average. All in all, it is clear both theoretically and empirically that the truthteller group probe-irrelevant RT difference mean will always be approximately the same (namely, zero). Because of this, the majority of published RT-CIT studies do not collect control data, but merely use probe-irrelevant difference means of different liar groups in order to compare them to each other and determine which ones are superior (only 11 out of 24 RT-CIT studies that we found contained at least one experiment that collected original control data; see the next section, Collection of Meta-Data).

Another set of studies also did not collect control data, but instead used computer simulations to produce control data by randomly drawing values from normally distributed values with a mean of zero [24–26]. One approach was based on the simulation of individual responses [26]. This particular simulation procedure involved standardizing individual probe and irrelevant responses (i.e., calculating standard scores, also called z-scores, normal scores, etc.), in cases of both truthtellers and liars (details below). This evaluation approach, in turn, was based on a similar procedure widely used in evaluating electrodermal responses, which yields superior results due to the specific nature of electrodermal activity [27]. It was this procedure based on which another paper [28] introduced, for their electrodermal response-based CIT, the control data simulation approach that has subsequently often been cited in relation to RT-CIT studies too [26].

The main point here is that the standardization procedure leads to a different predictor than the conventional probe-irrelevant difference, and there is no known evidence that the standardization has any sort of advantage in case of the RT-CIT. It is a wholly different question whether the related simulation approach for the standardized predictors is valid or not. Therefore, before addressing the matter of data simulation, we first evaluate whether or not the probe value standardized based on mean responses to probe and irrelevant items may be a more efficient predictor than the mean probe-irrelevant difference.

Furthermore, for completeness, we also consider another method using standardization, namely, probe-irrelevant difference standardized based on trial-level responses [29, 30]. This was introduced (following an algorithm used for Implicit Association Test evaluations [31]) as an attempt to find a reasonable cutoff point in the absence of control data which would allow the calculation of an optimal cutoff [30]. By calculating the probe-irrelevant difference as uncorrected Cohen’s *d*, the authors reasoned that such an individual effect size may follow the conventional interpretation of small, moderate, and large for *d* = 0.2, 0.5, or 0.8 [30]. Based on their empirical finding that a cutoff at 0.2 results in an optimal balance between specificity and sensitivity in their experiment, it was subsequently used in several experiments to set cutoff point for providing participants with immediate „caught or not” feedback following the test [24, 32, 33]. Some studies have also used this individual effect size, instead of the conventional mean differences, to calculate AUCs [29, 33–35]. There has been, however, no attempt to show whether or not this measure indeed provides a more generalizable cutoff than simple mean differences.

In sum, we have three different possible procedures for calculating a predictor variable, none of which had yet been statistically compared to each other under any consideration in the RT-CIT: (a) the mean of all response times to probes minus the mean of all response times for irrelevants [23], (b) probe value standardized based on mean responses to probe and irrelevant items [26–28], and (c) probe-irrelevant difference standardized based on trial-level responses [30].

For the evaluation of these procedures in classification efficiency per study as well as in the generalizability of cutoff points, we collected all available empirical control and liar data from studies where both of these conditions were included. We subsequently used the same empirical data to devise and test a new approach for the simulation of control data. Therefore, in the following, we first describe our meta data collection, then the evaluation of classification efficiency and the generalizability of cutoff points, and finally, we outline and validate our new simulation approach.

## Collection of meta-data

The literature search and inclusion criteria largely followed a recent meta-analysis [15], except for two important additional restrictions: we included only CIT paradigms with primary focus on RT measurement, and only studies that collected truthteller data along with liar data.

We searched the electronic data-bases PsycINFO, Web of Science, and PubMed, using the following search string: ("Guilty knowledge" OR "Concealed information" OR GKT OR CIT OR "guilty action" OR "memory detection" OR "Concealed knowledge" OR "decept*" OR "lie detection" OR "lie-detection" OR "decei*") AND ("Reaction time*" OR "Response time*" OR "Response latenc*"). Within the found articles, references of relevant articles as well as subsequent citations (via Google Scholar) were searched for potential further articles to be included.

The following criteria were used to select studies for inclusion:

- The study was an experimental study reporting original data.
- The study sample consisted of adults or the mean age of the sample was at least 18 years.
- The study reported results of a computer-based study using a CIT paradigm, where RT was the primary measure. We did not include studies that focused on other primary measures (e.g., ERPs, fMRI).
- The study emphasized deception or information concealment through instructions.
- Raw data for all trials in each individual test had to be available. If no such data was available, the authors were contacted to provide the data. If this data could not be provided, the study was excluded.

The databases were searched on September 28, 2019. The search resulted in 1,832 findings, and, after removing all duplicates, 1,561 unique references (S1 File). Screening based on titles and abstracts, 1,534 references were excluded primarily because they were not deception detection related, or otherwise because they did not report original data, did not use the CIT paradigm, or were not primarily RT-based. Out of the remaining 24 studies, 11 contained at least one experiment in which original truthteller data was collected. Four studies had publicly available data (or were made available upon request [24, 29, 33, 35]. For studies with no publicly available data, the authors of the papers were contacted, and thereby we obtained the data from three further studies [30, 34, 36]. The authors of the remaining five studies told us that the data is no longer available (despite efforts to retrieve it and also partial aggregated data from one case [23, 37–40].

Thus, our final sample drew data from seven different studies, including 12 experimental CIT designs (i.e., separate methods tested), each with both liar and control data, between which the key between-condition effect sizes were calculated. Note that each of the main meta-analytic comparisons in the present paper compare two different measures within each of these 12 group pairs. Hence, for each of these main comparisons there were 12 against 12 effect sizes to compare within-study.

For completeness we also report the overall average effect sizes as well as other potential moderators unrelated to our paper. These latter results could be affected by publication bias as well as selection bias (due to unavailable data), and therefore certainly not intended to provide any reliable insight into the RT-CIT in general, and neither do we draw any related conclusions in our paper.

## Method

For all calculations we followed the exclusion criteria of the latest related papers: an accuracy rate for the main items (probes and irrelevants merged) lower than 75%, and accuracy rate for target items lower than 50%. (In case of filler items: accuracy rate for either familiar- or unfamiliar-referring items lower than 50%.) For all further analysis, responses below 150 ms and were excluded, along with all trials with incorrect key response and too slow response (above 800 ms; uniform response deadline in all studies).

The standardized probe value based on mean responses to probe and irrelevant items [26–28] was calculated as follows: First, the mean RT was calculated for each individual item included in the test (e.g., in case of birthdays, one mean RT for all responses for trials with the text “AUG 25,” similarly one mean RT for “JAN 12,” etc., regardless of whether they are probes or irrelevants). Second, the obtained mean value for the probe item was standardized relative to all the probe and irrelevant mean values (i.e., the mean of all values was subtracted from the probe value, and the resulting value was divided by the standard deviation of all values). In mathematical notation (with *P* for probe RT mean, *M*_*all*_ for the mean of all probe and irrelevant RT means, *SD*_*all*_ for the standard deviation of all probe and irrelevant RT means): $\frac{P-M_{all}}{{SD}_{all}}$. The standardized probe item value was then used as the final predictor variable. (In case of multiple probe items in an individual test, the standardized probe item values were averaged to obtain a single predictor value.) This predictor will be referred to as *standardized probe RT*.

The probe-irrelevant difference standardized based on trial-level responses was calculated as the uncorrected Cohen’s *d* between all probe and all irrelevant RTs [30]. Namely, the difference between the probe RT mean and the irrelevant RT mean (which is the conventional *mean probe-irrelevant [RT] difference*) was divided by the pooled standard deviation of probe RTs and irrelevant RTs, using all trials. In mathematical notation (with *M* for arithmetic mean, *n* for sample number, *pr* for all probe RTs, and *ir* for all irrelevant RTs): $\frac{M_{pr}-M_{ir}}{\sqrt{\frac{{(n}_{pr}-1)({{SD}_{pr}}^{2})+{(n}_{ir}-1)({{SD}_{ir}}^{2})}{{n_{pr}+n}_{ir}-2}}}$. This predictor will be referred to as *standardized probe-irrelevant (RT) difference*.

All data processing, statistical tests, and plots were written in R [41–45] (S2 File). Wherever possible, we report Bayes factor (BFs) as a complementary statistic that may be of interest to some readers. Our interpretation however always relies on the well-established frequentist interference, using the conventional alpha level of .05.

## Different predictors: Descriptives and diagnostic efficiency

Statistics from the meta-data most relevant to the present paper are all presented in Tables 1, 2, and 3.

**Table 1:** Means and standard deviations of predictors.

Study reference	#	N_L_	N_C_	MPID				SPRT				SPID			
				Liar		Control		Liar		Control		Liar		Control	
				M	SD	M	SD	M	SD	M	SD	M	SD	M	SD
Geven, Ben–Shakhar, et al. (2018) MP ^a^	1	242	38	32.6	35.2	4.9	25.5	0.68	0.63	0.13	0.59	0.29	0.30	0.05	0.22
Kleinberg & Verschuere (2015) Exp1 MP	2	88	118	34.9	36.5	5.2	24.6	0.53	0.48	0.08	0.41	0.28	0.28	0.04	0.20
Kleinberg & Verschuere (2015) Exp2 MP	3	103	113	32.8	36.5	0.0	26.6	0.52	0.52	0.01	0.47	0.29	0.35	0.00	0.23
Kleinberg & Verschuere (2016) Exp1 MP	4	159	97	34.6	32.4	–5.2	24.1	0.65	0.52	–0.08	0.50	0.30	0.28	–0.03	0.20
Kleinberg & Verschuere (2016) Exp2 MP	5	193	53	27.7	38.5	2.3	27.5	0.48	0.62	0.00	0.48	0.21	0.28	0.01	0.23
Lukács, Kleinberg, et al. (2017) Exp1 MP	6	81	75	27.4	26.3	4.0	19.8	0.55	0.48	0.08	0.37	0.26	0.26	0.03	0.17
Lukács, Kleinberg, et al. (2017) Exp1 SP	7	98	80	8.8	19.9	–0.8	18.6	0.19	0.44	–0.02	0.39	0.07	0.17	–0.01	0.15
Lukács, Kleinberg, et al. (2017) Exp1 SPF	8	45	49	38.6	31.4	0.6	18.1	0.72	0.53	0.03	0.41	0.38	0.32	0.00	0.17
Noordraven & Verschuere (2013) MP ^a^	9	17	20	71.5	43.6	9.2	23.1	0.83	0.52	0.13	0.36	0.66	0.41	0.10	0.25
Verschuere & Kleinberg (2015) MP ^a^	10	40	31	52.7	25.4	–2.2	15.2	0.60	0.21	–0.08	0.28	0.45	0.22	–0.02	0.13
Verschuere, Kleinberg, et al. (2015) Exp2 MP	11	44	49	56.8	43.4	8.0	29.5	0.76	0.54	0.13	0.46	0.46	0.36	0.07	0.25
Verschuere, Kleinberg, et al. (2015) Exp2 SP	12	52	64	22.9	34.4	–2.6	29.3	0.31	0.47	–0.05	0.43	0.17	0.26	–0.02	0.23
	*M*			36.8	33.6	1.9	23.5	0.57	0.50	0.03	0.43	0.32	0.29	0.02	0.20

Means (*M*) and standard deviations (*SD*) for the differently calculated Predictors obtained from empirical data. *MPID*: mean probe-irrelevant difference (ms); *SPRT*: standardized probe RT, and *SPID*: standardized probe-irrelevant difference. Next to the study reference, the protocol types are indicated as *SP*: single-probe, *MP*: multiple-probe, *SPF*: single-probe with familiarity-related filler items. Liar and Control (i.e. truthteller) sample sizes are shown under the headers *N*_*L*_ and *N*_*C*_. The values below the number sign [*#*] header are numbered labels for each experimental design, for easier subsequent reference. The last row *M* shows the means (unweighted) of all values from the corresponding column above.

^a^ Offline data collection (i.e., participants not crowdsourced).

**Table 2:** Detection rates and thresholds per predictor.

#	MPID					SPRT					SPID				
	Optimal		Inferred		Thres.	Optimal		Inferred		Thres.	Optimal		Inferred		Thres.
	TP	TN	TP	TN		TP	TN	TP	TN		TP	TN	TP	TN	
1	.60	.84	.59	.84	18.8	.72	.68	.70	.68	0.29	.62	.84	.60	.84	0.17
2	.86	.58	.65	.75	7.1	.84	.63	.65	.73	0.15	.61	.83	.64	.76	0.21
3	.74	.71	.58	.78	10.8	.71	.71	.60	.75	0.22	.70	.75	.57	.79	0.12
4	.74	.82	.67	.85	15.3	.66	.87	.72	.78	0.49	.73	.80	.65	.86	0.12
5	.57	.75	.53	.77	15.8	.56	.77	.58	.74	0.35	.61	.75	.50	.77	0.12
6	.59	.88	.59	.83	20.9	.58	.88	.65	.72	0.47	.64	.83	.58	.87	0.15
7	.59	.69	.26	.89	5.4	.56	.69	.37	.80	0.16	.47	.80	.21	.86	0.11
8	.87	.78	.71	.86	12.0	.84	.80	.80	.80	0.28	.84	.78	.69	.84	0.11
9	.82	.90	.82	.70	43.4	.82	.80	.82	.80	0.38	.82	.85	.82	.70	0.35
10	.82	.00	.88	.90	29.6	.85	.97	.82	.97	0.31	.82	.00	.88	.87	0.27
11	.64	.92	.80	.57	45.3	.59	.92	.80	.65	0.70	.66	.84	.77	.67	0.30
12	.63	.72	.44	.81	11.7	.63	.72	.44	.88	0.16	.62	.72	.40	.88	0.09
*M*	.71	.80	.63	.80	19.70	.70	.79	.66	.77	0.33	.68	.82	.61	.81	0.18

True positive (*TP*) and true negative (*TN*) rates, using Optimal or Inferred thresholds, for the differently calculated Predictors, from empirical data. *MPID*: mean probe-irrelevant difference; *SPRT*: standardized probe RT, and *SPID*: standardized probe-irrelevant difference. The thresholds (*Thres*.) shown are the optimal ones used for each study. (To note, the inferred thresholds are not depicted, since they are very similar for each study due to their calculation as the average optimal threshold of all other studies: see the last row *M*, which shows the means [unweighted] of all values from the corresponding column above.) For the experiment names and details referenced here only by the numbers [#], see Table 1.

**Table 3:** Effect sizes and areas under the curves.

#	MPID_real_		MPID_sim_		SPRT		SPID	
	ES	AUC	ES	AUC	ES	AUC	ES	AUC
1	0.81	.75	0.96	.78	0.88	.74	0.83	.74
2	0.98	.77	1.15	.80	1.04	.77	1.03	.78
3	1.04	.77	1.06	.77	1.03	.77	1.01	.77
4	1.35	.85	1.19	.81	1.42	.84	1.32	.84
5	0.70	.70	0.77	.72	0.81	.72	0.73	.70
6	1.00	.77	1.17	.80	1.09	.78	1.04	.79
7	0.50	.64	0.48	.63	0.50	.63	0.49	.63
8	1.50	.86	1.44	.84	1.47	.85	1.52	.87
9	1.83	.88	2.04	.91	1.59	.85	1.69	.86
10	2.54	.97	2.33	.96	2.79	.98	2.51	.97
11	1.33	.83	1.59	.87	1.26	.81	1.26	.81
12	0.81	.71	0.80	.69	0.80	.71	0.77	.70
*M*	1.20	.79	1.25	.80	1.22	.79	1.18	.79

Effect sizes (*ES*) and areas under the curves (*AUC*), for the differently calculated Predictors. *MPID*: mean probe-irrelevant difference; *SPRT*: standardized probe RT, and *SPID*: standardized probe-irrelevant difference. Both *SPRT* and *SPID* are obtained from empirical data. In case of *MPID*, the ES and AUC obtained using simulated data is also depicted: *MPID*_*real*_ refers to the original statistics from empirical data, while *MPID*_*sim*_ refers to the simulated statistics. The last row *M* shows the means (unweighted) of all values from the corresponding column above. For the experiment names and details referenced here only by the numbers [#], see Table 1.

Most clearly, Table 1 shows that the overall average empirical control mean probe-irrelevant difference hardly differs from zero (1.9 ms, SD = 23.5). In contrast, the overall average empirical liar probe-irrelevant difference is 36.8 ms, SD = 33.6. These numbers may prove useful for future studies to estimate how the outcomes compare to previous outcomes (although keeping in mind potential moderating differences, e.g., most clearly, the given CIT protocol). The alternative predictors (standardized probe RT, standardized probe-irrelevant difference) differ nominally but are analogous (i.e., close to zero in case of controls, magnitudes larger in case of liars).

Tables 2 and 3 show diagnostic values. Correct detection rates (true positive and true negative), depicted in Table 2, most straightforwardly reflect diagnostic accuracy. Crucially, optimal detection rates, based on the Youden’s index of each given study (for which the detection rates are calculated), are somewhat higher than the inferred detection rates, based on the mean Youden’s index of all studies except for the given study (for which the detection rates are calculated; S1 Appendix). However, this difference is moderate (ca. 3–4% overall for any of the predictors), and in particular remarkably small in case of true negative rates (in fact identical up to two digits for mean probe-irrelevant difference). Both optimal and inferred detection rates are similar in case of any of the predictor types, indicating no advantage in either accuracy or generalizability of one predictor over any other (see S1 Appendix).

Finally, Table 3 depicts AUCs and effect sizes. Again, different predictors lead to very similar outcomes (e.g., AUCs identical up to the two digits shown in the table), hence once again no particular advantage appears for any of the predictors. Importantly, however, simulated values are also very close to empirical values for mean probe-irrelevant differences, suggesting that our simulation procedure (described below) is highly accurate.

### Statistical comparison of predictors

We statistically evaluated the two RT-CIT predictor calculations as alternatives to the conventional mean probe-irrelevant difference in a study-level comparison to assess whether the different predictors chosen influence the liar-truthteller effect size (which in turn reflects classification efficiency) or the related AUCs. We subsequently evaluated whether any of these approaches would lead to more generalizable cut-off points in comparison to the mean probe-irrelevant difference, also with a more general aim to assess if a generalizable cutoff point for the RT-CIT can be established. All related analysis is reported in detail in S1 Appendix. In brief, most importantly, the alternative predictors did not show any significant benefit in any respect (and in fact result in very similar outcomes in all cases), as reflected in Tables 2 and 3. Therefore, for the main analyses and demonstrations in the rest of the manuscript we use only the conventional mean probe-irrelevant differences, for simplicity. Nonetheless, all findings below hold equally true for either of the two alternative predictors as well.

## Classification efficiency as opposed to mean differences

The main argument of the present paper is that mean differences of the mean probe-irrelevant differences between two methods are not strictly indicative of classification efficiency. Namely, when a given lie detection method has larger mean probe-irrelevant differences than another method, but it also has larger SD (in either liar or control data or both), it may not be more efficient in classification.

Here we illustrate this in simulations (S2 Script) based on the collected empirical meta-data. In each of these simulations, we used a normally distributed sample of a 1,000 hypothetic mean probe-irrelevant difference values, for two group pairs of liar and control data. (That the empirical control data is generally normally distributed is supported by the density graphs and Q-Q plots per each of the 12 empirical datasets, see S3 File. The S2 Script also includes demonstration that the slight positive skewness, as seen in some empirical data, has very little impact.) The simulated control data always had a mean of zero. The average of the two simulated liar data means was always 36.79 ms: This number is the mean of all empirical probe-irrelevant means in our study (Table 1). The outcomes vary somewhat when modifying this average: When the liar data means are larger, the liar data values are generally further from the control group data values, and hence the overlap is less affected by the SD of either group. Nonetheless, the principle remains the same.

**Fig 2:**
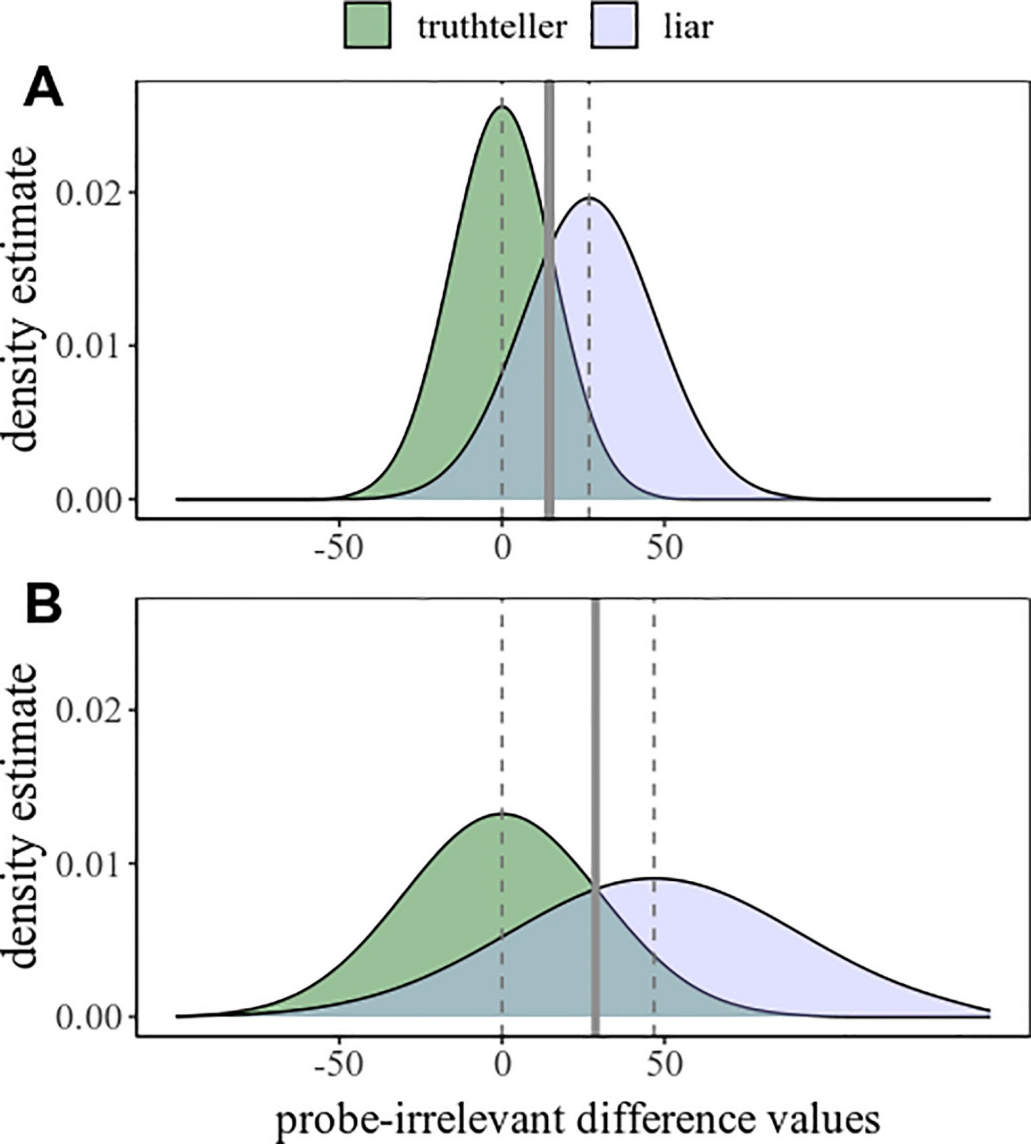
Density plots of probe-irrelevant differences. In truthteller and liar conditions, for two simulated scenarios, method A and B, using, for SDs, the minimum and maximum SDs taken from the meta-data. Solid vertical lines indicate optimal cutoff points for classification, while dashed vertical lines indicate the mean values of the given liar and truthteller groups.

In the first simulation, we use the extremes of SDs found in our empirical meta-data, and a mean difference (between mean probe-irrelevant differences) of 20 ms. Thereby, method A with lower probe-irrelevant differences had a simulated liar dataset with a mean of 26.79 and an SD of 19.87 (smallest SD among all empirical liar datasets), and a simulated control dataset with a mean of zero and an SD of 15.24 (smallest SD among all empirical control datasets); while method B with higher probe-irrelevant differences had a simulated liar dataset with a mean of 46.79 and an SD of 43.64 (largest SD among all empirical liar datasets), and a simulated control dataset with a mean of zero and an SD of 29.48 (largest SD among all empirical control datasets); see Fig 2. Due to the smaller SDs in method A, despite the larger probe-irrelevant means in method B, the AUC in method A (.858) is larger than the AUC in method B (.813).

In this specific scenario, the effect size between the mean probe-irrelevant differences of the two methods is *d* = 0.59, which is a large effect size that is not uncommon to be found in RT-CIT studies when comparing two different methods to demonstrate a significant difference. This means that in this specific case, the statistic may be wrongly taken as proof for that method B is more efficient than method A, while in fact the very opposite is true. All other parameters remaining equal in this scenario, a minimum difference of around 26.5 ms (*d* = 0.79) between the two liar means is required for method B to have a nominally higher AUC then method A; namely, .830 over .827 –which is still of hardly any practical relevance.

**Fig 3:**
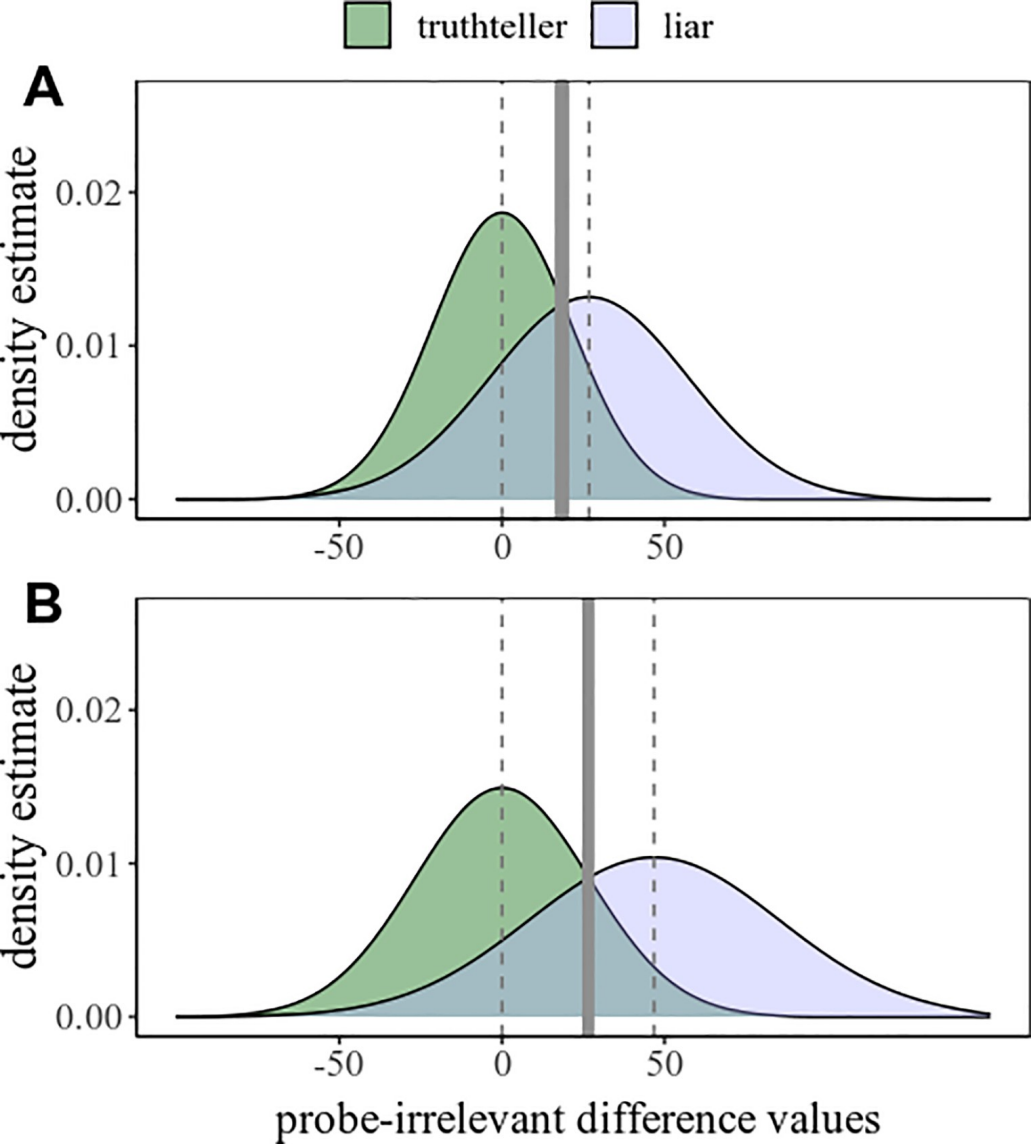
Density plots of probe-irrelevant differences. In truthteller and liar conditions, for two simulated scenarios, method A and B, using, for SDs, the lower and upper limits of the 95% CI of the SDs taken from the meta-data. Solid vertical lines indicate optimal cutoff points for classification, while dashed vertical lines indicate the mean values of the given liar and truthteller groups.

Nonetheless, taking the most extreme possible SDs found in our meta-data is a far-fetched example. A more realistic worst-case scenario may be constructed by using the upper and lower 95% CI limits of the empirical SDs from the respective conditions. We again took a mean difference of 20 ms, hence only the SDs changed: Method A had a simulated liar dataset with a mean of 26.79 and an SD of 29.60, and a simulated control dataset with a mean of zero and an SD of 20.89; while method B with higher probe-irrelevant differences had a simulated liar dataset with a mean of 46.79 and an SD of 37.63, and a simulated control dataset with a mean of zero and an SD of 26.12; see Fig 3.

In this case, method B indeed shows a larger AUC (.847) than method A (.771). With these settings, the effect size difference between the liar groups is *d* = .59, similar to the first example. Method A will have larger AUC only when the mean difference is at least as low as 8 ms, with *d* = 0.24 (in which case the AUC is .818 for method A, and .814 for method B). Such a small effect size has never been found to be significant in RT-CIT studies, since no study has ever had a large enough sample size to detect it: For example, for a paired *t*-test (to minimize required sample), for a power of .90, alpha of .05, the required sample would be 184 participants. An analogous independent *t*-test would require 730 participants– 366 per group. Even using crowdsourcing, it is unlikely that future studies would invest in gathering so many participants for a single comparison of two methods.

All in all, we may conclude that, in general practice, when significant mean differences in mean probe-irrelevant differences are found (between two liar groups), the classification efficiency is also at least to some extent improved for the method with larger mean probe-irrelevant differences. However, (a) it cannot be ruled out that extreme cases may occur where this is entirely untrue, and (b) even if it is true, the difference can be substantially over- or underestimated, resulting in misleading implications about practical relevance.

This could imply that it is best to always collect control data in RT-CIT studies and compare the obtained AUCs. However, there are two problems with this. First, calculating power for the comparison of two AUCs is complicated: There is no widely accepted method and different approaches can yield very different results [46–49]. Second, the required sample would be generally multiple times larger than for the comparison of means. For example, take an ideal case where methods A and B have the same SD for both liars (33 ms; average SD from the meta-data) and controls (23 ms; average SD from the meta-data), and again the 20 ms difference. In this case, the effect size between liar groups is a large *d* = 0.61. For a paired *t*-test comparison, for a power of .9, alpha of .05, the required sample would be only 32 participants. For a paired AUC comparison of the same data, for the same power and alpha, the required sample size would already be slightly more than the double: 33 liar and 33 control participants (calculated for DeLong’s test [50]; some alternative methods can result in much larger required samples [51, 52]). However, when the SDs differ even a little bit in the direction that decreases the AUC differences (i.e., larger SD for the method with larger probe-irrelevant differences), the AUC requires increasingly larger samples. For example, if we modify the previous example to subtract just 3 ms from the SD of the results of one method (with smaller probe-irrelevant differences) and add just 3 ms to the other’s, the required sample size will be 90 (45 liars and 45 controls). Now, a mean difference of 10 ms with all other settings unchanged would require 119 participants for a paired *t*-test (*d* = 0.30), but 420 for AUC comparison (210 liars and 210 controls; and, again, alternative methods for power calculation can result in even much larger numbers).

As described in the introduction, control data may be simulated to spare resources [24, 26, 28], but, to the best of our knowledge, such simulation procedures have never been empirically validated.

## Computer simulation of control data

### Empirical dispersion

Previous simulation procedures had various implicit assumptions (i.e., unstated but necessary for the validity of the given procedure) about the SDs: (a) equal SDs in all experiments and designs when using standardized probe RT, reproducible by a specific randomized procedure [26], (b) control data SD equal to liar data SD [25], or (c) equal control data SDs in similar experimental settings [24]. It is logical that similar experiments lead to similar SDs, but the extent of similarity required would be difficult to estimate or test empirically. In any case, often there is no precedent of similar experimental settings. The other two assumptions–(a) and (b)–can be easily tested.

First (a), following the procedure of [26, 28], we obtained control data per “participant” by drawing five random values from normal distribution as mean RT per item, randomly assigning one value as “probe” item mean RT, and subsequently calculating a standardized probe RT as described previously in case of empirical data (i.e., subtracting the mean of all five items and dividing by the SD of all five items). To present a good estimate of the resulting control data SD, using this procedure we generated, 1000 times, a 100 control participants’ simulated data (S3 Script).

The mean of the SDs from these 1000 simulated datasets is 0.894, 95% CI [0.891, 0.897]. This means that when control data is simulated using this procedure, the SD of the standardized probe RT values will always be around 0.894. However, as can be seen in Table 1, the empirical control group SDs (for standardized probe RTs) are notably lower than this in every study in our meta-data (with a mean of 0.429, 95% CI [0.384, 0.473]). A one sample *t*-test, comparing the empirical control group SDs to the theoretical mean of 0.894, gives very strong evidence for the difference; *t*(11) = 20.37, *p* < .001, *BF*_10_ = 1.7 ×10^7^ (raw mean difference: 0.43, 95% CI [0.38, 0.48]). This means, as demonstrated in the previous section, that this simulation procedure would always substantially underestimate AUCs in RT-CIT studies. Furthermore, once again, this procedure presupposes the use of standardized probe RTs as the predictor variable in the RT-CIT, even though it does not appear to be superior to the conventional mean probe-irrelevant difference.

Second (b), to test whether empirical control data SDs equal to empirical liar data SDs, we compared the two datasets with a *t*-test, which showed, with strong evidence, that liar data SDs are larger; *t*(11) = 7.25, *p* < .001, *d* = 2.09, 95% CI [1.05, 3.11], *BF*_10_ = 1399.14 (raw mean difference: 10.1, 95% CI [7.0, 13.2]). Therefore, simulated truthteller data SD based on empirical liar data SD would be overestimated (i.e. assumed to have more widely dispersed data than in real empirical truthteller cases), which in turn means that the AUCs would be underestimated in this case too.

**Fig 4:**
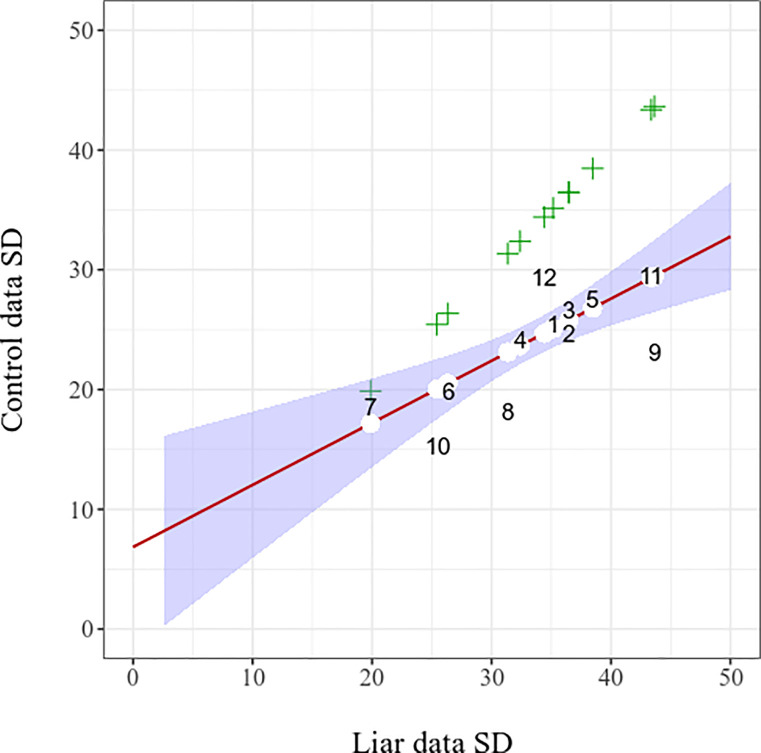
Empirical and derived control data Standard Deviations (SDs) in function of liar data SDs. The number labels in the figure are the datasets (individual experimental designs) in the alphabetical order of their full name, as in Table 1, showing the empirical control data SD for each empirical liar data SD. Green crosses show presumed control data SDs equaling liar SDs for each given study. White filled circles show the control SDs based on fitted values for each study in the weighted linear regression model. Hence, for each dataset, there are three vertically aligned values indicated: empirical data (study number), hypothetical control SD equaling empirical liar SD (cross), and hypothetical fitted SD value (white circle). The red line shows the slope of the weighted regression, along with the surrounding 95% CI in light blue.

It is however true that control data SDs are significantly and strongly *correlated* with liar data SDs; *r*(10) = .738, 95% CI [.284, .921] (*R*^2^ = .544), *p =* .006, *BF*_10_ = 7.06; see Fig 4. To take the samples sizes into account, we repeated the correlation test but now weighted by the sample sizes of each experimental design (liar and control group together); the evidence and the correlation were then even stronger; *r*(10) = .826, 95% CI [.476, 1] (*R*^2^ = .682), *p =* .002.

Consequently, one may use this correlation to predict control data SDs based on empirical liar data SDs, namely, by building a linear regression model and using the fitted values for the simulated control data SDs. We entered the full sample size of each experimental design as weights for the regression. The intercept was at 6.86, while the coefficient of liar data SDs was 0.52. Thereby, for any given study, the control data SD may be calculated by multiplying the liar data SD by 0.52 and adding 6.86 (i.e., *SD*_*liar*_ × 0.52 + 6.86; which gives the corresponding fitted value in the regression model; see white circles in Fig 4).

### Evaluation of simulated data

To evaluate the procedure, we used a cross-validated procedure in which simulated control data for each experimental design was generated, using near-perfect normal distribution, with a mean of zero and the SD calculated based on a weighted regression model *without* the data from the given experimental design [53–55]. That is, we fitted 12 separate models as described above, except for that in each case we left out the data of a single experimental design. The intercept and coefficients obtained from each of these models were then used to calculate the SD for the data simulation for the given experimental design that was left out. The SD was then calculated, in each case, as the given design’s liar data SD multiplied by the coefficient, plus the intercept, from the given corresponding model. To prevent unbalanced weight in contributing to the pooled variances (from liar and control data), each simulated control data contained the same number of values as the corresponding design’s empirical control data. All comparisons below concern the simulated data obtained using the described leave-one-out procedure versus the empirical control data.

The main question here was whether the effect between liar and control groups differs when using simulated control data as opposed to empirical control data. Therefore, this factor was the main moderator to be tested in the following meta-analysis.

We included two moderators that were likely to influence the effect size, although they are not theoretically relevant to the present paper (for details, see S1 Appendix). Firstly, CIT *Protocol*: single-probe (SP) protocol, multiple-probe (MP) protocol, or single-probe protocol with familiarity-related filler items (SPF) [24, 25, 35, 56]. Secondly, *Crowdsourcing*: crowdsourced (online) experiment or laboratory experiments [57, 58]. While there is no minimal number of studies for conducting meta-analysis [59, 60], the relatively small number of included studies is very unlikely to provide reliable and generalizable evidence for these potential moderating influences–as noted before, we draw no related conclusions.

Thus, we ran a random-effects model with the following factors as potential moderators: Simulation (Yes vs. No), Protocol (SP, MP, SPF), Crowdsourcing (Yes vs. No). The random effects model indicated a meta-analytic effect of 1.58, 95% CI [1.20, 1.96]. The model showed a significant effect of the moderators *Q*_*M*_(4) = 15.97, *p* = .003. Nonetheless, the residual heterogeneity was still significant, *Q*_*E*_(19) = 65.06, *p <* 0.001, indicating that our moderators cannot fully explain all heterogeneity among the studies.

The key result is that the Simulation moderator was not significant; *B =* 0.05, 95% CI [–0.26, 0.35], *p =* .759 (simulated effect sizes nominally slightly larger, but CIs indicate that an effect size difference larger than 0.35 is very unlikely [61]).

The Protocol effect was significant, *Q*_*M*_(2) = 7.38, *p* = .025. Pairwise follow-up comparisons showed that SP had smaller effects than MP or SPF; *B* = 0.46, 95% CI [0.04, 0.87], *p =* .030, *B* = 0.83, 95% CI [0.17, 1.49], *p =* .014; and there was no significant difference between SPF and MP, *B* = 0.37, 95% CI [–0.22, 0.96], *p =* .217. The Crowdsourcing effect was significant, with smaller effects in crowdsourced studies, *B* = 0.51, 95% CI [0.10, 0.91], *p* = .013.

**Fig 5:**
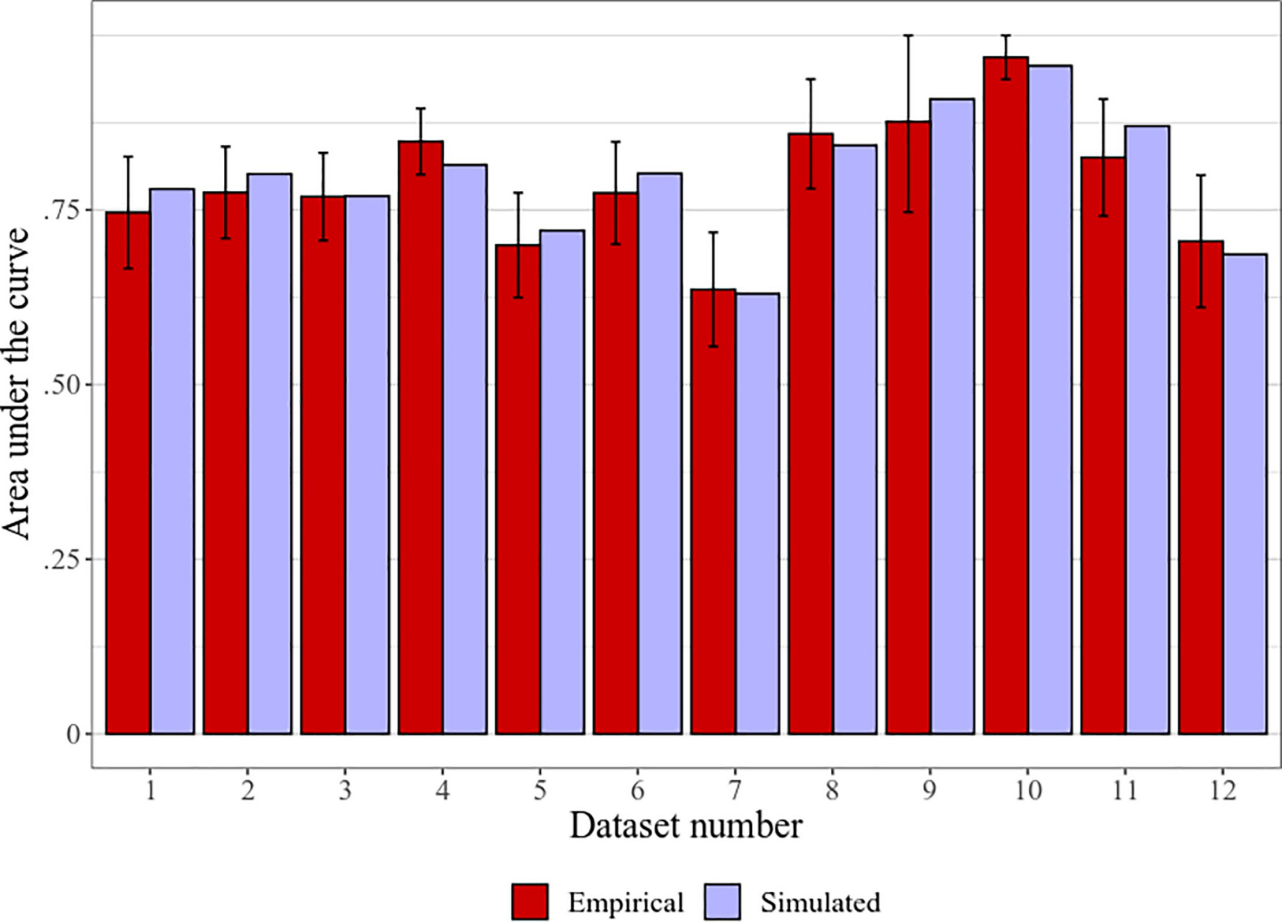
Areas under the curves using empirical or simulated control data. Means with 95% CIs in error bars. Datasets are individual experimental designs designated with numbers in the alphabetical order of their full name, as in Table 1.

As a supplementary test for AUCs in specific, we compared the obtained AUC values with a *t*-test. That is, we generated the simulated data as described above for the meta-analysis, and calculated AUCs for classification between liar data and empirical control data, as well as liar data and simulated control data. The test showed no significant difference, *t*(11) = 1.14, *p =* .278, *d* = 0.33, 95% CI [–0.26, 0.90], *BF*_01_ = 2.03. The simulated AUCs were on average only nominally larger (*M*±*SD* = .799±.092 vs. .790±.091), with a raw mean difference of .008, 95% CI [–.008, .025]; see Fig 5.

Crucially, the correlation between the empirical and simulated AUCs is extremely high: *r*(10) = .961, 95% CI [.862, .989] (*R*^2^ = .923), *p* < .001, BF_10_ = 830.84. This means that it is highly unlikely that there will be a substantial difference between the simulated AUCs of any two specific methods if there is no difference between the empirical AUCs. Consequently, AUCs simulated using this procedure may be used to verify differences demonstrated by comparison of liar predictor means.

In fact, some–but perhaps even most–of the chance difference between the empirical and simulated data can be explained by error in the empirical data. Since in the control condition probe and irrelevant items were assigned completely randomly, the difference between them must always converge to zero. That is, with a large enough (or infinite) sample that cancels out random noise, this difference will always be zero. However, there is always some random variation in empirical control data that leads to probe-irrelevant differences that are small and statistically nonsignificant, but may still nominally influence between-condition effect sizes and AUCs. For example, in the first experiment by Kleinberg & Verschuere (2016 Exp. 1; dataset #4), the probe means were somewhat smaller than irrelevant means (*d* = 0.11), hence the mean probe-irrelevant differences were biased in the negative direction (–5.2±24.1 ms; see Table 1), and thereby introduced a small positive bias in the between-condition effect size and AUC (as compared to the AUC using a theoretical larger sample with zero probe-irrelevant differences). As seen in Table 3 and Fig 5 (dataset #4), the simulated effect size and AUC (using a control data mean of zero) is slightly smaller, and presumably more precise. Conversely, one of the designs in the Verschuere et al. (MP; 2015; dataset #11) had notably larger probe means than irrelevant means (*d* = 0.14; probe-irrelevant differences: 8.0±29.5 ms), thereby decreasing between-condition effect size and AUC–which are somewhat larger, and probably more precise, when using the simulated data (Table 1 and Fig 5; dataset #11). Tellingly, all 12 datasets, with no exception, follow the very same pattern (i.e., negatively and positively biased empirical control predictors result in, respectively, smaller and larger simulated outcomes for both effect sizes and AUCs). Therefore, from this perspective, the simulated data can actually be assumed to more precisely reflect real outcomes than a empirical control data of limited sample size.

## Conclusions and recommendations

The main points that we demonstrated in this paper are that (1) dispersion matters: comparing mean differences is not strictly indicative of classification efficiency, and (2) a reliable estimation of classification efficiency can be generated using a simple simulation procedure.

With regard to point (1), the differences between probe and irrelevant means and the related within-test probe-irrelevant effect sizes are regularly used to assess, informally or formally, the efficiency of different lie detection methods, even in comparisons across entirely different studies, paradigms, and technologies, and consequently, based on these comparisons, authors draw important practical conclusions [15, 62–66]. As we have shown here, such conclusions cannot be directly drawn. Comparison of liar probe-irrelevant differences should always be complemented by the addition of a control data.

This control data, however, may be simulated based on the dispersion of liar data, which brings us to point (2). Based on 12 real empirical control datasets, we devised and tested a simulation procedure that proved to be highly accurate. In S4 Script, we provide a demonstration of how this simulation may be performed in an instant using a few simple lines of code. The AUCs with simulated control data can be used to verify that the dispersion has no substantial influence.

Nonetheless, such simulated data cannot perfectly equal empirical data in all scenarios, and we still encourage the collection of empirical control data whenever sufficient resources are available. In the future, such new empirical data may also be used to recalculate (and reverify) the optimal simulations procedure.

### Power calculation

Prospective (a priori) power analysis to determine the required sample size is crucial for behavioral experiments [67]. To determine the smallest effect size of interest required for the power analysis, the ideal way is to rely on objective justification [68–70]. In case of the CIT, one may consider the sample size required for a reasonable increase in the rate of correct detections of liars and truthtellers in relation to the costs of the required sample size. However, when planning the collection of two groups of liar participants whose mean probe-irrelevant RT differences are to be directly compared to each other, the effect size (standardized mean difference) has no direct implication for the practical consequences in diagnostics. Unsurprisingly, no RT-CIT study to date has reported any objective (i.e., diagnostics-related) justification for sample sizes. Practical consequences can however be estimated a priori using the very same simulation procedure described in the present paper: Diagnostic efficiency values (e.g., correct detection rates and areas under the curves) can be simulated for any given expected effect sizes between liar conditions alone. An online Shiny R implementation for such estimations is freely available via https://github.com/gasparl/esdi (including detailed documentation and usage examples specific to RT-CIT).

### Data sharing

As a beneficial corollary of our study, we also provide a neatly arranged data file of raw trials from 2265 individual tests (including 8001 separate probes, from 12 different experimental designs, all involving both liar and truthteller conditions), which can be directly used for further large volume exploratory analyses, such as gauging the effects of overlapping visual features (e.g., probe starts with same letter as target), passing of time (e.g., probe-irrelevant differences in function of trial number), assessing potential model-based predictors combining various variables (e.g., error rates, target RT, etc.), or repeating our analyses for independent replication or to answer further related questions.

On a related note, we would like to urge authors to always upload their raw trial-level (and possibly also aggregated) data, along with a clear description when needed, to a publicly available repository to allow for studies like ours and the ones outlined above to be conducted [71]. This is also in the interest of the original authors if they want to keep track of their own data, which may be otherwise difficult to locate and decipher later on. We also see a role here for reviewers, who in the peer review process can ensure that the data is indeed fully available. This should not only help future efforts as described above but also help the reviewers more accurately assess the quality of the study under review [72, 73].

## Summary

In the present paper we showed that previously suggested alternative predictor calculations have no particular benefit in the RT-CIT, and relatedly demonstrated that cutoff points, while fairly generalizable, are not stable across different experiments. Based on empirical data, we demonstrated that comparing mean differences is not strictly indicative of classification efficiency, and we devised and validated procedure for generating control data to simulate AUCs. Incidentally, we also shared the vast dataset–used for our analyses–of unified raw variables from a number of different experiments for further large-scale analyses.

## Supporting information

S1 Checklist PRISMA 2009 checklist. (PDF)

S1 Appendix Meta-analytical evaluation of different predictors. A detailed report of the evaluation of the three different potential predictors (mean probe-irrelevant difference; standardized probe; standardized probe-irrelevant difference) in respect of (a) classification efficiency of different predictors on study level, and (b) generalizability of cutoff points and related classification efficiency.
(PDF)

S1 Script R code for illustrative distributions and diagnostics. Figures and their underlying data as described in the Introduction.
(R) Click here for additional data file.

S2 Script R code for classification efficiency versus mean differences examples. Function to create plots and print results, as well as all examples given in the main text.
(R) Click here for additional data file.

S3 Script R code for to test previous simulation procedure based on standardized probe RT. Demonstrates that the control data simulation procedure used by Meijer et al. (2007) for electrodermal response-based CIT is not valid for RT-CIT.
(R) Click here for additional data file.

S4 Script R code for generating simulated control data. A short example code, including explanatory comments, for generating simulated control data using the procedure describe in the present paper.
(R) Click here for additional data file.

S1 File Literature search results. The lists of references obtained from the search, as well as after at each of the exclusion steps (either as extracted search results or as plain text).
(ZIP)

S2 File Data files and R codes for the main analyses. Full original raw dataset, extensive aggregated statistics, and all code used for meta-analytic and other comparisons using the data.
(ZIP)

S3 File Density and Q-Q plots per datasets. Figures for the assessment of normal distribution of empirical liar as well as control (truthteller) predictor values (individual probe-irrelevant RT mean differences) in each of the 12 datasets.
(ZIP)

## References

[1] Fiedler K, Schmid J, Stahl T. What is the current truth about polygraph lie detection? Basic and Applied Social Psychology. 2002;24: 313–324. 10.1207/S15324834BASP2404_6

[2] Lykken DT. A tremor in the blood: uses and abuses of the lie detector. New ed. New York: Plenum Trade; 1998.

[3] Granhag PA, Vrij A, Verschuere B, editors. Detecting Deception: Current Challenges and Cognitive Approaches. Chichester, UK: John Wiley & Sons, Ltd; 2014 10.1002/9781118510001

[4] National Research Council. Polygraph and lie detection. Washington, D.C.: The National Academies Press; 2003 Available: http://www.nap.edu/openbook.php?record_id=10420

[5] Verschuere B, Ben-Shakhar G, Meijer E. Memory detection: theory and application of the concealed information test. Cambridge: Cambridge University Press; 2011.

[6] Green DM, Swets JA. Signal detection theory and psychophysics. Huntington, N.Y: R. E. Krieger Pub. Co; 1974.

[7] Zou KH, O’Malley AJ, Mauri L. Receiver-Operating Characteristic Analysis for Evaluating Diagnostic Tests and Predictive Models. Circulation. 2007;115: 654–657. 10.1161/CIRCULATIONAHA.105.594929 17283280

[8] Sternglanz RW, Morris WL, Morrow M, Braverman J. A Review of Meta-Analyses About Deception Detection In: Docan-Morgan T, editor. The Palgrave Handbook of Deceptive Communication. Cham: Springer International Publishing; 2019 pp. 303–326. 10.1007/978-3-319-96334-1_16

[9] Franz VH, von Luxburg U. No Evidence for Unconscious Lie Detection: A Significant Difference Does Not Imply Accurate Classification. Psychol Sci. 2015;26: 1646–1648. 10.1177/0956797615597333 26302983

[10] Kleinberg B, Arntz A, Verschuere B. Being accurate about accuracy in verbal deception detection. Capraro V, editor. PLoS ONE. 2019;14: e0220228 10.1371/journal.pone.0220228 31393894 PMC6687387

[11] Youden WJ. Index for rating diagnostic tests. Cancer. 1950;3: 32–35. 10.1002/1097-0142(1950)3:1&lt;32::aid-cncr2820030106&gt;3.0.co;2-3 15405679

[12] DeLong ER, DeLong DM, Clarke-Pearson DL. Comparing the areas under two or more correlated receiver operating characteristic curves: A nonparametric approach. Biometrics. 1988;44: 837 10.2307/2531595 3203132

[13] Rice ME, Harris GT. Comparing effect sizes in follow-up studies: ROC Area, Cohen’s d, and r. Law and Human Behavior. 2005;29: 615–620. 10.1007/s10979-005-6832-7 16254746

[14] Manikandan S. Measures of dispersion. Journal of Pharmacology and Pharmacotherapeutics. 2011;2: 315 10.4103/0976-500X.85931 22025871 PMC3198538

[15] Suchotzki K, Verschuere B, Van Bockstaele B, Ben-Shakhar G, Crombez G. Lying takes time: A meta-analysis on reaction time measures of deception. Psychological Bulletin. 2017;143: 428–453. 10.1037/bul0000087 28182460

[16] Meijer EH, Selle NK, Elber L, Ben-Shakhar G. Memory detection with the Concealed Information Test: A meta analysis of skin conductance, respiration, heart rate, and P300 data: CIT meta-analysis of SCR, respiration, HR, and P300. Psychophysiology. 2014;51: 879–904. 10.1111/psyp.12239 24916920

[17] Lasko TA, Bhagwat JG, Zou KH, Ohno-Machado L. The use of receiver operating characteristic curves in biomedical informatics. Journal of Biomedical Informatics. 2005;38: 404–415. 10.1016/j.jbi.2005.02.008 16198999

[18] Obuchowski NA. Receiver Operating Characteristic Curves and Their Use in Radiology. Radiology. 2003;229: 3–8. 10.1148/radiol.2291010898 14519861

[19] Zweig MH, Campbell G. Receiver-operating characteristic (ROC) plots: a fundamental evaluation tool in clinical medicine. Clin Chem. 1993;39: 561–577. 8472349

[20] Kharin VV, Zwiers FW. On the ROC Score of Probability Forecasts. JOURNAL OF CLIMATE. 2003;16: 6.

[21] Swets JA. Signal detection theory and ROC analysis in psychology and diagnostics: Collected papers. Hillsdale, NJ, US: Lawrence Erlbaum Associates, Inc; 1996.

[22] Seymour TL, Schumacher EH. Electromyographic evidence for response conflict in the exclude recognition task. Cognitive, Affective, & Behavioral Neuroscience. 2009;9: 71–82. 10.3758/CABN.9.1.71 19246328

[23] Seymour TL, Seifert CM, Shafto MG, Mosmann AL. Using response time measures to assess “guilty knowledge.” J Appl Psychol. 2000;85: 30–37. 10.1037/0021-9010.85.1.30 10740954

[24] Lukács G, Kleinberg B, Verschuere B. Familiarity-related fillers improve the validity of reaction time-based memory detection. Journal of Applied Research in Memory and Cognition. 2017;6: 295–305. 10.1016/j.jarmac.2017.01.013

[25] Lukács G, Ansorge U. Information leakage in the response time‐based Concealed Information Test. Appl Cognit Psychol. 2019;33: 1178–1196. 10.1002/acp.3565

[26] Visu-Petra G, Varga M, Miclea M, Visu-Petra L. When interference helps: increasing executive load to facilitate deception detection in the concealed information test. Front Psychol. 2013;4: 146 10.3389/fpsyg.2013.00146 23543918 PMC3610081

[27] Ben-Shakhar G. Standardization Within Individuals: A Simple Method to Neutralize Individual Differences in Skin Conductance. Psychophysiology. 1985;22: 292–299. 10.1111/j.1469-8986.1985.tb01603.x 4011799

[28] Meijer EH, Smulders FTY, Johnston JE, Merckelbach HLGJ. Combining skin conductance and forced choice in the detection of concealed information. Psychophysiology. 2007;44: 814–822. 10.1111/j.1469-8986.2007.00543.x 17584188

[29] Kleinberg B, Verschuere B. Memory detection 2.0: the first web-based memory detection test. Ben Hamed S, editor. PLOS ONE. 2015;10: e0118715 10.1371/journal.pone.0118715 25874966 PMC4395266

[30] Noordraven E, Verschuere B. Predicting the sensitivity of the reaction time-based Concealed Information Test. Applied Cognitive Psychology. 2013;27: 328–335. 10.1002/acp.2910

[31] Greenwald AG, Nosek BA, Banaji MR. Understanding and using the Implicit Association Test: I. An improved scoring algorithm. Journal of Personality and Social Psychology. 2003;85: 197–216. 10.1037/0022-3514.85.2.197 12916565

[32] Geven LM, Ben-Shakhar G, Kindt M, Verschuere B. It’s a match!? Appropriate item selection in the Concealed Information Test. Cogn Research. 2019;4: 11 10.1186/s41235-019-0161-8 30945051 PMC6447635

[33] Verschuere B, Kleinberg B. ID-check: online Concealed Information Test reveals true identity. Journal of Forensic Sciences. 2015;61: S237–S240. 10.1111/1556-4029.12960 26390033

[34] Kleinberg B, Verschuere B. The role of motivation to avoid detection in reaction time-based concealed information detection. Journal of Applied Research in Memory and Cognition. 2016;5: 43–51. 10.1016/j.jarmac.2015.11.004

[35] Verschuere B, Kleinberg B, Theocharidou K. RT-based memory detection: Item saliency effects in the single-probe and the multiple-probe protocol. Journal of Applied Research in Memory and Cognition. 2015;4: 59–65. 10.1016/j.jarmac.2015.01.001

[36] Geven LM, Klein Selle N, Ben-Shakhar G, Kindt M, Verschuere B. Self-initiated versus instructed cheating in the physiological Concealed Information Test. Biol Psychol. 2018;138: 146–155. 10.1016/j.biopsycho.2018.09.005 30236614

[37] Hu X, Evans A, Wu H, Lee K, Fu G. An interfering dot-probe task facilitates the detection of mock crime memory in a reaction time (RT)-based concealed information test. Acta Psychologica. 2013;142: 278–285. 10.1016/j.actpsy.2012.12.006 23376139

[38] Seymour TL, Fraynt BR. Time and Encoding Effects in the Concealed Knowledge Test. Applied Psychophysiology and Biofeedback. 2009;34: 177–187. 10.1007/s10484-009-9092-3 19536648 PMC2727398

[39] Seymour TL, Kerlin JR. Successful detection of verbal and visual concealed knowledge using an RT-based paradigm. Applied Cognitive Psychology. 2008;22: 475–490. 10.1002/acp.1375

[40] Visu-Petra G, Miclea M, Visu-Petra L. Reaction time-based detection of Concealed Information in relation to individual differences in executive functioning. Applied Cognitive Psychology. 2012;26: 342–351. 10.1002/acp.1827

[41] Kelley K. MBESS: The MBESS R package. R package version 4.5.1. 2019. Available: https://CRAN.R-project.org/package=MBESS

[42] Makowski D, Ben-Shachar M, Lüdecke D. bayestestR: Describing effects and their uncertainty, existence and significance within the Bayesian framework. JOSS. 2019;4: 1541 10.21105/joss.01541 PMC6914840 31920819

[43] Morey RD, Rouder JN. BayesFactor: Computation of Bayes factors for common designs. R package version 0.9.12–4.2. 2018. Available: https://CRAN.R-project.org/package=BayesFactor

[44] R Core Team. R: A language and environment for statistical computing. R Foundation for Statistical Computing, Vienna, Austria 2019 Available: https://www.R-project.org/

[45] Viechtbauer W. Conducting Meta-Analyses in *R* with the metafor Package. J Stat Soft. 2010;36 10.18637/jss.v036.i03

[46] Demler OV, Pencina MJ, D’Agostino RB. Misuse of DeLong test to compare AUCs for nested models. Statist Med. 2012;31: 2577–2587. 10.1002/sim.5328 22415937 PMC3684152

[47] Hajian-Tilaki K. Sample size estimation in diagnostic test studies of biomedical informatics. Journal of Biomedical Informatics. 2014;48: 193–204. 10.1016/j.jbi.2014.02.013 24582925

[48] Obuchowski NA. ROC Curves in Clinical Chemistry: Uses, Misuses, and Possible Solutions. Clinical Chemistry. 2004;50: 1118–1125. 10.1373/clinchem.2004.031823 15142978

[49] Obuchowski NA, McClish DK. Sample size determination for diagnostic accuracy studies involving binormal ROC curve indices. Stat Med. 1997;16: 1529–1542. 10.1002/(sici)1097-0258(19970715)16:13&lt;1529::aid-sim565&gt;3.0.co;2-h 9249923

[50] Robin X, Turck N, Hainard A, Tiberti N, Lisacek F, Sanchez J-C, et al pROC: an open-source package for R and S+ to analyze and compare ROC curves. BMC Bioinformatics. 2011;12: 77 10.1186/1471-2105-12-77 21414208 PMC3068975

[51] Goksuluk D, Korkmaz S, Zararsiz G, Karaagaoglu A Ergun. easyROC: An Interactive Web-tool for ROC Curve Analysis Using R Language Environment. The R Journal. 2016;8: 213 10.32614/RJ-2016-042

[52] Obuchowski NA. ROC Analysis. American Journal of Roentgenology. 2005;184: 364–372. 10.2214/ajr.184.2.01840364 15671347

[53] Hastie T, Tibshirani R, Friedman JH. The elements of statistical learning: data mining, inference, and prediction. 2nd ed New York, NY: Springer; 2009.

[54] Lachenbruch PA, Mickey MR. Estimation of Error Rates in Discriminant Analysis. Technometrics. 1968;10: 1–11. 10.1080/00401706.1968.10490530

[55] Riley RD, Ahmed I, Debray TPA, Willis BH, Noordzij JP, Higgins JPT, et al Summarising and validating test accuracy results across multiple studies for use in clinical practice: Summarising and validating test accuracy results across multiple studies for use in clinical practice. Statist Med. 2015;34: 2081–2103. 10.1002/sim.6471 25800943 PMC4973708

[56] Eom J-S, Sohn S, Park K, Eum Y-J, Sohn J-H. Effects of Varying Numbers of Probes on RT-based CIT Accuracy. International Journal of Multimedia and Ubiquitous Engineering. 2016;11: 229–238. 10.14257/ijmue.2016.11.2.23

[57] Chandler J, Paolacci G, Peer E, Mueller P, Ratliff KA. Using Nonnaive Participants Can Reduce Effect Sizes. Psychol Sci. 2015;26: 1131–1139. 10.1177/0956797615585115 26063440

[58] Zhou H, Fishbach A. The pitfall of experimenting on the web: How unattended selective attrition leads to surprising (yet false) research conclusions. Journal of Personality and Social Psychology. 2016;111: 493–504. 10.1037/pspa0000056 27295328

[59] Pigott TD, editor. Advances in meta-analysis. New York, NY: Springer; 2012.

[60] Valentine JC, Pigott TD, Rothstein HR. How Many Studies Do You Need?: A Primer on Statistical Power for Meta-Analysis. Journal of Educational and Behavioral Statistics. 2010;35: 215–247. 10.3102/1076998609346961

[61] Lakens D. Equivalence tests: A practical primer for *t* tests, correlations, and meta-analyses. Social Psychological and Personality Science. 2017;8: 355–362. 10.1177/1948550617697177 28736600 PMC5502906

[62] Georgiadou K, Chronos A, Verschuere B, Sauerland M. Reaction time-based Concealed Information Test in eyewitness identification is moderated by picture similarity but not eyewitness cooperation. Psychological Research. 2019 [cited 12 Feb 2019]. 10.1007/s00426-018-1139-8 30635707 PMC9470627

[63] Sauerland M, Wolfs ACF, Crans S, Verschuere B. Testing a potential alternative to traditional identification procedures: Reaction time-based concealed information test does not work for lineups with cooperative witnesses. Psychological Research. 2017 [cited 12 Feb 2019]. 10.1007/s00426-017-0948-5 29181584 PMC6647190

[64] Suchotzki K, De Houwer J, Kleinberg B, Verschuere B. Using more different and more familiar targets improves the detection of concealed information. Acta Psychologica. 2018;185: 65–71. 10.1016/j.actpsy.2018.01.010 29407246

[65] Suchotzki K, Kakavand A, Gamer M. Validity of the reaction time Concealed Information Test in a prison sample. Frontiers in Psychiatry. 2019;9 10.3389/fpsyt.2018.00745 30728785 PMC6351463

[66] Verschuere B, Crombez G, Degrootte T, Rosseel Y. Detecting concealed information with reaction times: Validity and comparison with the polygraph. Applied Cognitive Psychology. 2010;24: 991–1002. 10.1002/acp.1601

[67] Perugini M, Gallucci M, Costantini G. A Practical Primer To Power Analysis for Simple Experimental Designs. International Review of Social Psychology. 2018;31: 20 10.5334/irsp.181

[68] Lakens D. Calculating and reporting effect sizes to facilitate cumulative science: a practical primer for t-tests and ANOVAs. Frontiers in Psychology. 2013;4 10.3389/fpsyg.2013.00863 24324449 PMC3840331

[69] Lakens D, Scheel AM, Isager PM. Equivalence Testing for Psychological Research: A Tutorial. Advances in Methods and Practices in Psychological Science. 2018;1: 259–269. 10.1177/2515245918770963

[70] Cohen J. Statistical power analysis for the behavioral sciences. 2nd ed Hillsdale, N.J: L. Erlbaum Associates; 1988.

[71] Wicherts JM, Borsboom D, Kats J, Molenaar D. The poor availability of psychological research data for reanalysis. American Psychologist. 2006;61: 726–728. 10.1037/0003-066X.61.7.726 17032082

[72] Martone ME, Garcia-Castro A, VandenBos GR. Data sharing in psychology. American Psychologist. 2018;73: 111–125. 10.1037/amp0000242 29481105 PMC5920518

[73] Wicherts JM, Bakker M, Molenaar D. Willingness to Share Research Data Is Related to the Strength of the Evidence and the Quality of Reporting of Statistical Results. Tractenberg RE, editor. PLoS ONE. 2011;6: e26828 10.1371/journal.pone.0026828 22073203 PMC3206853

