# Supplementary material for: Dispersion matters: Diagnostics and control data computer simulation in Concealed Information Test studies
Source: PLoS One. 2020 Oct 2;15(10):e0240259. doi: 10.1371/journal.pone.0240259 (PMC7531802; doi:10.1371/journal.pone.0240259)
Supplement: S1 Appendix — A detailed report of the evaluation of the three different potential predictors (mean probe-irrelevant difference; standardized probe; standardized probe-irrelevant difference) in respect of (a) classification efficiency of different predictors on study level, and (b) generalizability of cutoff points and related classification efficiency. (PDF) [file pone.0240259.s002.pdf]

S1 Appendix

Classification Efficiency of Different Predictors on Study Level

The main question here was whether the effect between empirical liar and empirical control groups is affected by the different calculation versions of the predictor variable. Hence, predictor version was the main moderator to be tested in the following meta-analysis.

We chose a random-effects model rather than a fixed-effects model because a fixed-effects model assumes homogeneity in the estimated effect sizes. Since effect sizes are likely to be influenced by the many differences in study features among the studies included, it is unlikely that the assumption of homogeneity would be met in our case. However, since we have hypotheses on which features of the study would influence the effect, we included two further moderators in the meta-analysis – although neither is theoretically relevant to the present paper. First, most clearly, there are three different CIT protocols used in the studies: single-probe (SP) protocol, multiple-probe (MP) protocol, and single-probe protocol with familiarity-related filler items (SPF). (Note that there is only one study with single-probe protocol with filler items . While there is no minimal number of studies for conducting meta-analysis, this is of course very limited evidence. In any case, again, this question not relevant to the present paper.) These protocol differences have been repeatedly shown to significantly affect RT-CIT outcomes. Second, we included the potential moderator of using crowdsourced (online) experiment as opposed to laboratory experiments. While there are dozens of studies confirming the validity of crowdsourced experiments, there is also some evidence that effect sizes can be reduced or biased in certain cases. Furthermore, crowdsourced RT-CIT studies have been using less salient probe items than laboratory studies, which can also strongly affect outcomes. Consequently, it is also important to note that we do not aim to assess the validity of crowdsourced RT-CIT studies (which may be the subject of future research, using direct comparisons). This potential moderator merely represents the overall difference

between crowdsourced and laboratory RT-CIT studies as they have been conducted so far, including any accompanying design or settings.

Thus, we ran a random-effects model, with the following factors as potential moderators: Predictor (mean probe-irrelevant difference, standardized probe RT, and standardized probe-irrelevant difference), Protocol (SP, MP, SPF), Crowdsourcing (Yes vs. No); see Table 1-3. The random effects model indicated a meta-analytic effect of 1.57, 95% CI [1.23, 1.91]. The model showed a significant effect of the moderators $Q_M(5) = 22.77$, $p < .001$. Nonetheless, the residual heterogeneity was still significant $Q_E(30) = 106.55$, $p < .001$, indicating that our moderators cannot fully explain all heterogeneity among the studies.

Both the Predictor and Protocol factors had more than two levels, therefore we first assessed if these factors were significant overall. Most importantly, the Predictor had no significant effect; $Q_M(2) = 0.060$, $p = .970$(the nominal differences were, as compared to mean probe-irrelevant differences, larger for standardized probe RT, with regression coefficient $B = 0.03$, 95% CI [–0.28, 0.34]; smaller for standardized probe-irrelevant difference: $B = –0.01$, 95% CI [–0.32, 0.30]; these two compared to each other: $B = 0.04$, 95% CI [–0.27, 0.35].

Less importantly, the Protocol effect was significant as expected, $Q_M(2) = 28.75$, $p = .005$. Pairwise follow-up comparisons showed that SPF had larger effects than SP ; $B = 0.86$, 95% CI [0.31, 1.41], $p = .002$; MP had also larger effect than SP, $B = 0.44$, 95% CI [0.09, 0.78], $p = .013$; and there was no significant difference between SPF and MP (despite a tendency for larger effect for SPF), $B = 0.42$, 95% CI [–0.07, 0.91], $p = .094$. The Crowdsourcing effect was also significant, with the expected smaller effects in crowdsourced studies, $B = 0.51$, 95% CI [0.17, 0.84], $p = .003$.

As a supplementary test for AUCs in specific, we compared the obtained AUC values across the three predictor versions in a one-way Welch corrected ANOVA. The test showed

no significant difference, $F(2,22) = 0.49$, $p = .617$, $\eta_p^2 = .043$, 90% CI [0, .173], $\eta_G^2 < .001$, $BF_{01} = 3.85$.

Generalizability of Cutoff points and Related Classification Efficiency

Since the between-condition effect size and the AUC (calculated between the same two conditions) are directly related, any increase in between-condition effect sizes necessarily indicates an increase in AUCs (except when the AUC cannot be further improved). However, unlike the AUC, effect size is not limited by ceiling effect, and therefore is an optimal measurement to compare different designs or predictors. For example, two methods compared in strictly controlled laboratory conditions may both yield AUCs around 98-100%, hence their difference will be neither statistically significant, nor apparently substantial. However, the effect sizes may differ significantly, which implies that under less optimal real-life conditions, the method with the larger effect size in the laboratory study may provide substantially higher AUC in field settings. That is why our meta-analytical comparison of effect sizes are not only valid, but, from this point of view, preferable to AUC comparisons.

However, from effect sizes alone the true positive and false positive rates at given cutoff values cannot be inferred. Therefore, for the evaluation of the generalizability of cutoff points, we cannot compare effect sizes, but instead directly compare the true positive rates (TPRs) and false positive rates (FPRs) obtained in the different conditions. For this, we used a leave-one-out cross-validation across the studies. First, we calculated the optimal thresholds (based on Youden's index) in all individual experimental designs for the liar and control group pairs, and, for each design, calculated TPRs and FPRs using the given design's optimal cutoff value. Afterwards, for each design, we calculated TPRs and FPRs using, as inferred cutoff points, the mean of the optimal cutoff values of all other designs.

Using the obtained TPRs and FPRs, we ran a three-way repeated measures ANOVA with factors Cutoff (Optimal vs. Inferred) × Condition (TPR vs. FPR) × Predictor (mean probe-

irrelevant difference, standardized probe RT, and standardized probe-irrelevant difference); see Fig A1.

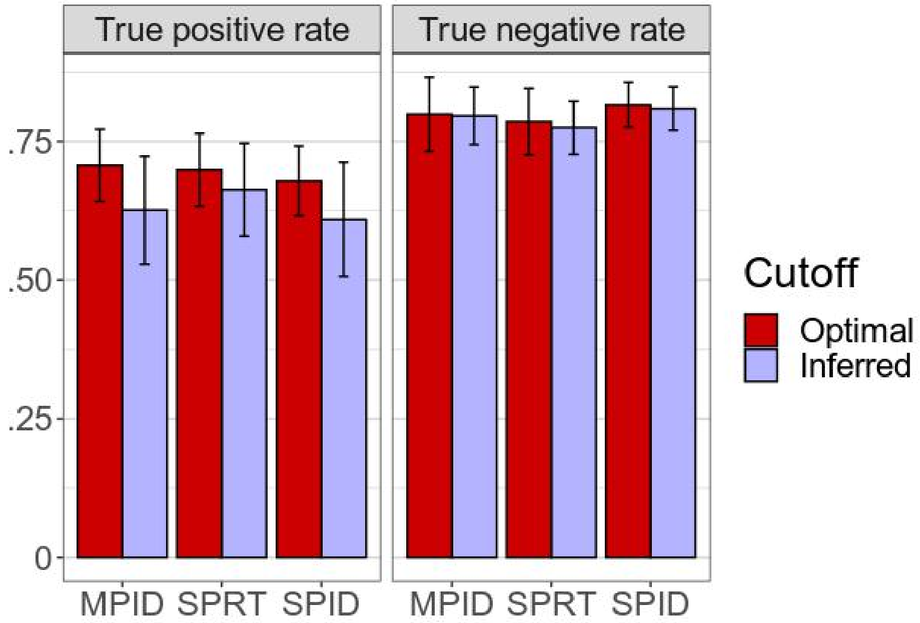

Fig A1. True positive and true negative rates, with Optimal and Inferred cutoff points, for the differently calculated Predictors.

Means with 95% CIs in error bars. *MPID*: mean probe-irrelevant difference; *SPRT*: standardized probe RT, and *SPID*: standardized probe-irrelevant difference.

The Cutoff main effect was significant, with larger accuracy rates for optimal cutoffs, $F(1,11) = 48.49$, $p < .001$, $\eta_p^2 = .815$, 90% CI [.546, .881], $\eta_G^2 = .022$, $BF_{10} = 1.62$. The Predictor main effect was not significant, $F(2,22) = 0.56$, $p = .580$, $\eta_p^2 = .048$, 90% CI [0, .184], $\eta_G2 < .001$, $BF_{01} = 14.00$. The Predictor × Cutoff and Predictor × Condition interactions were not significant; $F(2,22) = 2.39$, $p = .115$, $\eta_p^2 = .178$, 90% CI [0, .356], $\eta_G^2 = .001$, $BF_{01} = 7.44$; $F(2,22) = 3.22$, $p = .060$, $\eta_p^2 = .226$, 90% CI [0, .404], $\eta_G^2 = .015$, $BF_{01} = 2.54$; nor was the three-way interaction of Predictor × Condition × Cutoff; $F(2,22) = 0.54$, $p = .593$, $\eta_p^2 = .046$, 90% CI [0, .180], $\eta_G^2 = .002$, $BF_{01} = 5.22$. This means that the different predictor calculations did not affect the outcome when using inferred cutoff points; hence,

neither of the predictor alternatives proved superior to the conventional mean probe-irrelevant difference.

Finally, the Condition main effect was significant; $F(1,11) = 15.00$, $p = .003$, $\eta_p^2 = .577$, 90% CI [.179, .730], $\eta_G^2 = .250$, $BF_{10} = 1.41 \times 10^{\wedge 11}$; while the Condition × Cutoff interaction was not significant; $F(1,11) = 0.76$, $p = .403$, $\eta_p^2 = .064$, 90% CI [0, .328], $\eta_G^2 = .014$, $BF_{01} = 1.12$. This of course depends on how the inferred cutoff point is defined. For example, if we use medians instead of means in the cross-validation procedure, the inferred cutoffs will lead to somewhat higher TPRs and somewhat lower TNRs. Repeating an analogous ANOVA in this case, main results remain the same. (Cutoff: $F(1,11) = 78.19$, $p < .001$, $\eta_p^2 = .877$, 90% CI [.680, .920], $\eta_G^2 = .026$, $BF_{10} = 2.87$; Condition: $F(1,11) = 8.05$, $p = .016$, $\eta_p^2 = .423$, 90% CI [.054, .629], $\eta_G^2 = .144$, $BF_{10} = 1.32 \times 10^{\wedge 6}$; Predictor: $F(2,22) = 0.84$, $p = .443$, $\eta_p^2 = .071$, 90% CI [0, .224], $\eta_G2 < .001$, $BF_{01} = 14.18$; Condition × Cutoff: $F(1,11) = 0.26$, $p = .622$, $\eta_p^2 = .023$, 90% CI [0, .254], $\eta_G^2 = .005$, $BF_{01} = 2.52$; Cutoff × Predictor: $F(2,22) = 5.95$, $p = .009$, $\eta_p^2 = .351$, 90% CI [.064, .515], $\eta_G^2 = .002$, $BF_{01} = 7.44$; Condition × Predictor: $F(2,22) = 1.46$, $p = .254$, $\eta_p^2 = .117$, 90% CI [0, .287], $\eta_G^2 = .007$, $BF_{01} = 4.77$; Condition × Cutoff × Predictor: $F(2,22) = 0.09$, $p = .915$, $\eta_p^2 = .008$, 90% CI [0, .043], $\eta_G2 < .001$, $BF_{01} = 5.65$.)

Discussion

We evaluated two RT-CIT predictor calculations as alternatives to the conventional mean probe-irrelevant difference, but did not find either of them to have a better classification efficiency or more generalizable cutoff points (i.e., optimal cutoff points found in one study do not work better in other studies and individual cases when using alternative predictor calculation). It is understandable and well proven that the variance of electrodermal responses differs substantially between individuals and therefore such response values need to be standardized per each test, but in case of simple response times this does not appear to be true,

at least for the purpose of CIT evaluations. Nonetheless, in individual cases the standardized probe-irrelevant difference may be informative for researchers less familiar with the RT-CIT but familiar with Cohen's *d*.

In our analyses we actually also explored slightly different variations of the two alternative predictors (all calculations available in the S2 File). For one, we calculated standardized probe RT using trial-level standardization (i.e., we standardized trial level probe and irrelevant RTs and then took the mean of standardized probe values), considering that this might more closely reflect the reasoning behind the use of standardization for the continuous electrodermal measure (i.e., trial level RTs are more continuous than RTs aggregated per items). For another, we calculated standardized probe-irrelevant difference using, as denominator, the SD from irrelevant trials only (instead of the pooled SD for the regular uncorrected Cohen's d as standardized mean difference), because this was the calculation used in certain papers [33,35] (but not by Noordraven and Verschuere [30] who originally introduced this measure as regular uncorrected Cohen'd). Both these variations led to very similar results as their more conventional counterpart (as presented above), and in fact both gave nominally slightly smaller effect sizes and AUCs.

We have furthermore demonstrated, for the first time, the generalizability of cutoff points in the RT-CIT. This is important in cases when immediate individual evaluation is given in any new scenario where there is no sufficient data yet for the calculation of an optimal cutoff in the given settings – which can be a new experiment where immediate individual feedback is needed (e.g., for reward), as well as in future potential real life application of the RT-CIT. Unsurprisingly, the accuracy rates decreased when using cutoff points inferred from other studies instead of using the optimal cutoff points for each given dataset. However, while statistically significant, this decrease was moderate, and reflected primarily in TPR difference only – a reassuring finding in view of the arguable priority of

TNR, reflecting the protection of innocent subjects, over TPR (see Table 2: TPR .71±.11 and TNR .80±.12 for optimal cutoff, while TPR .63±.17 and TNR .80±.09 for inferred cutoff, for the conventional mean probe-irrelevant difference predictor). This implies that the cutoff points are fairly generalizable, and individual evaluation in novel scenarios is not much less reliable than those determined using the receiver operating characteristics of a previous sample of liars and truthtellers. The related statistics shown in Table 2 (along with those in Table 1 and Table 3) may serve as a useful future reference for RT-CIT researchers.

Altogether, we can conclude that the conventional mean probe-irrelevant difference is a good estimate of the effect and that generalizable cut off points can be found.
