# Supplementary figures and images for: Dispersion matters: Diagnostics and control data computer simulation in Concealed Information Test studies
Source: PLoS One. 2020 Oct 2;15(10):e0240259. doi: 10.1371/journal.pone.0240259 (PMC7531802; doi:10.1371/journal.pone.0240259)

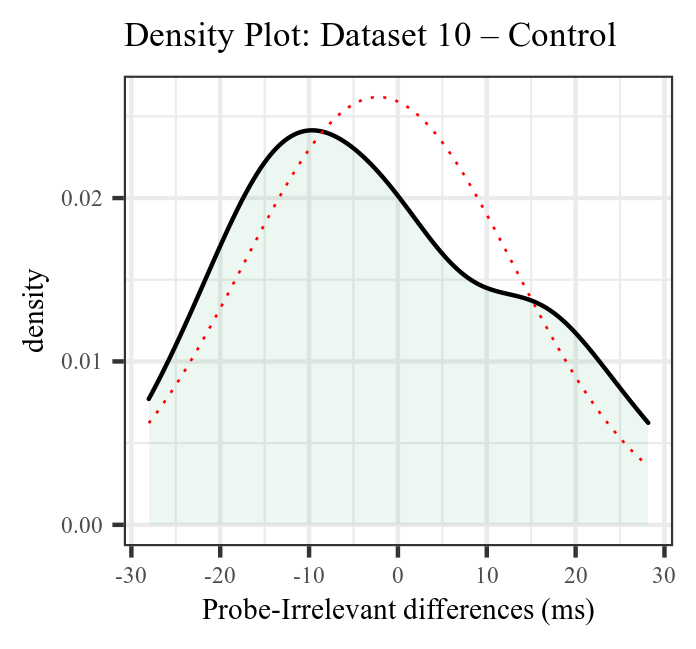

Supplement: S3 File — Figures for the assessment of normal distribution of empirical liar as well as control (truthteller) predictor values (individual probe-irrelevant RT mean differences) in each of the 12 datasets. (ZIP) [file pone.0240259.s009.zip › dataset_10_Control_density_plot.tiff]

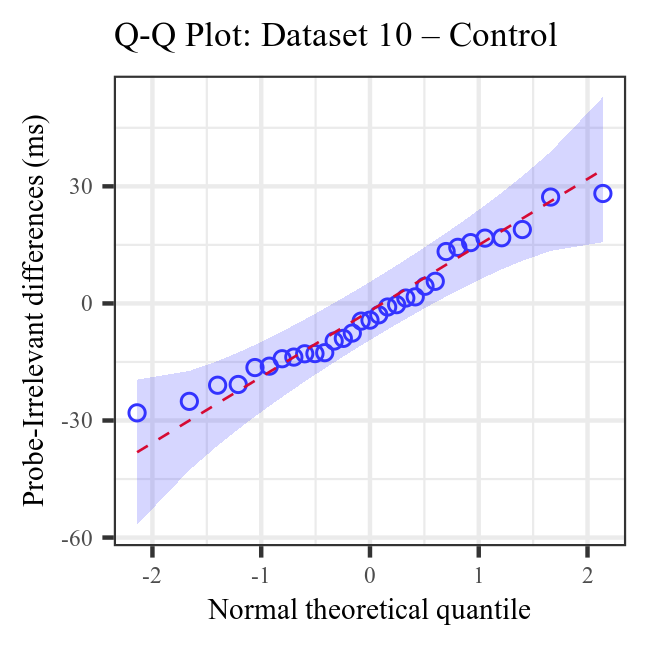

Supplement: S3 File — Figures for the assessment of normal distribution of empirical liar as well as control (truthteller) predictor values (individual probe-irrelevant RT mean differences) in each of the 12 datasets. (ZIP) [file pone.0240259.s009.zip › dataset_10_Control_qq_plot.tiff]

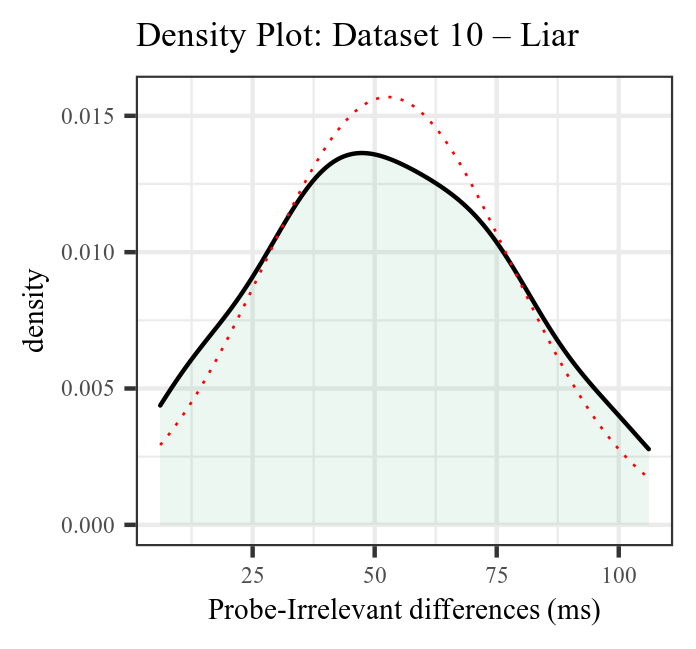

Supplement: S3 File — Figures for the assessment of normal distribution of empirical liar as well as control (truthteller) predictor values (individual probe-irrelevant RT mean differences) in each of the 12 datasets. (ZIP) [file pone.0240259.s009.zip › dataset_10_Liar_density_plot.tiff]

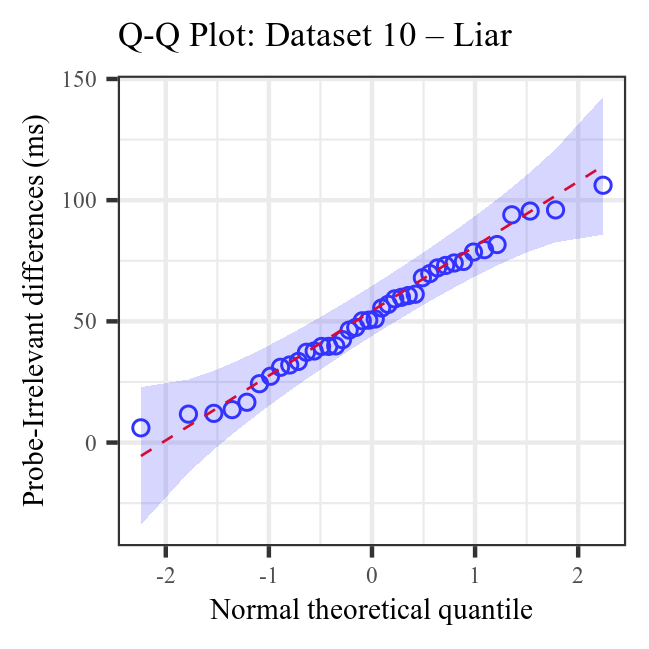

Supplement: S3 File — Figures for the assessment of normal distribution of empirical liar as well as control (truthteller) predictor values (individual probe-irrelevant RT mean differences) in each of the 12 datasets. (ZIP) [file pone.0240259.s009.zip › dataset_10_Liar_qq_plot.tiff]

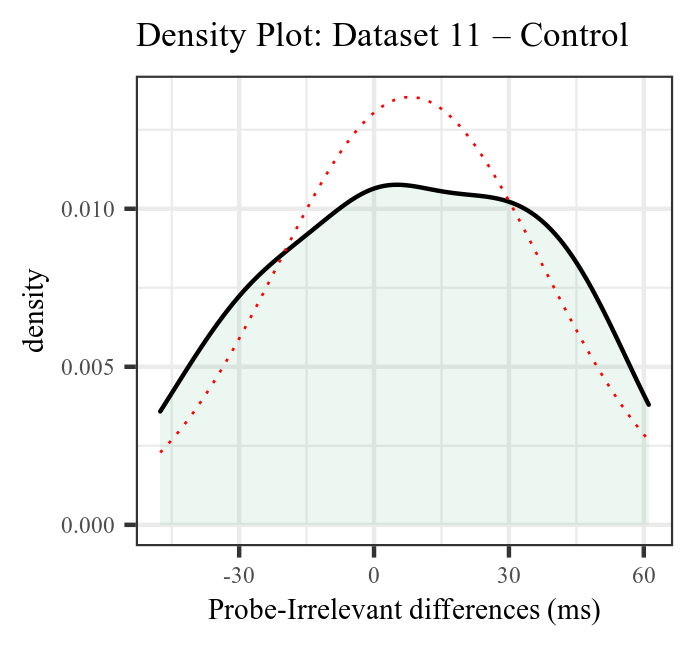

Supplement: S3 File — Figures for the assessment of normal distribution of empirical liar as well as control (truthteller) predictor values (individual probe-irrelevant RT mean differences) in each of the 12 datasets. (ZIP) [file pone.0240259.s009.zip › dataset_11_Control_density_plot.tiff]

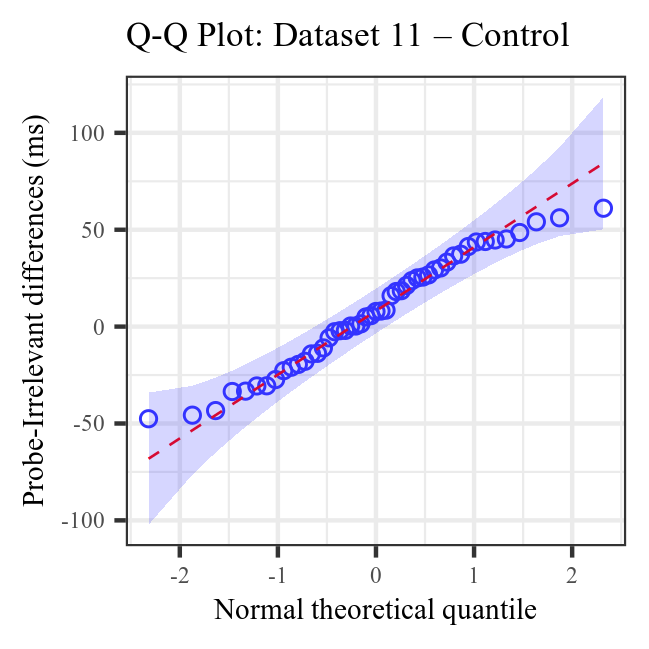

Supplement: S3 File — Figures for the assessment of normal distribution of empirical liar as well as control (truthteller) predictor values (individual probe-irrelevant RT mean differences) in each of the 12 datasets. (ZIP) [file pone.0240259.s009.zip › dataset_11_Control_qq_plot.tiff]

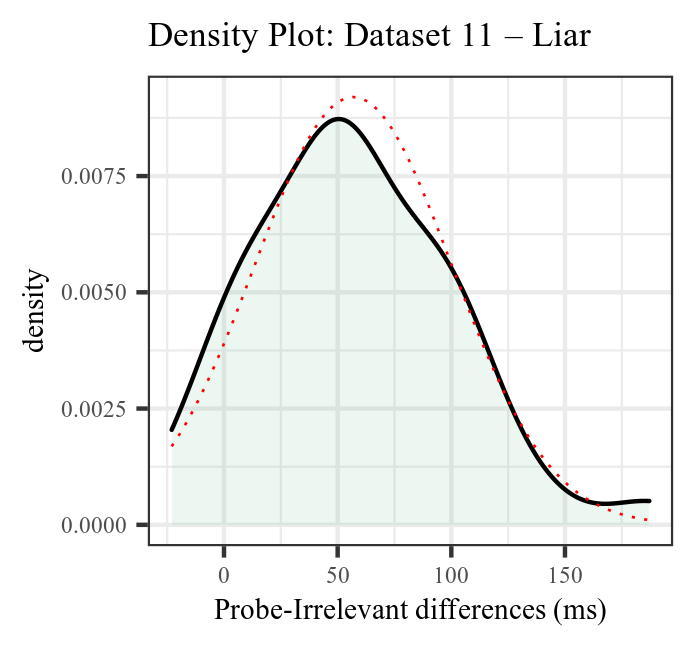

Supplement: S3 File — Figures for the assessment of normal distribution of empirical liar as well as control (truthteller) predictor values (individual probe-irrelevant RT mean differences) in each of the 12 datasets. (ZIP) [file pone.0240259.s009.zip › dataset_11_Liar_density_plot.tiff]

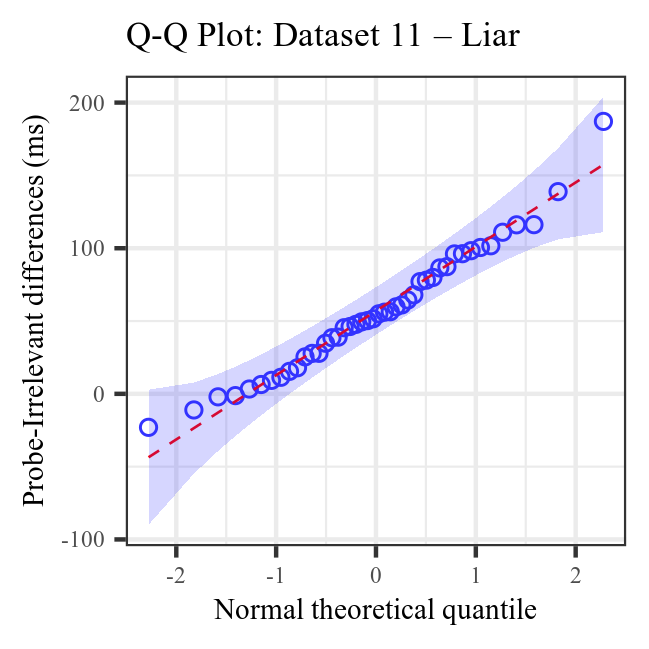

Supplement: S3 File — Figures for the assessment of normal distribution of empirical liar as well as control (truthteller) predictor values (individual probe-irrelevant RT mean differences) in each of the 12 datasets. (ZIP) [file pone.0240259.s009.zip › dataset_11_Liar_qq_plot.tiff]

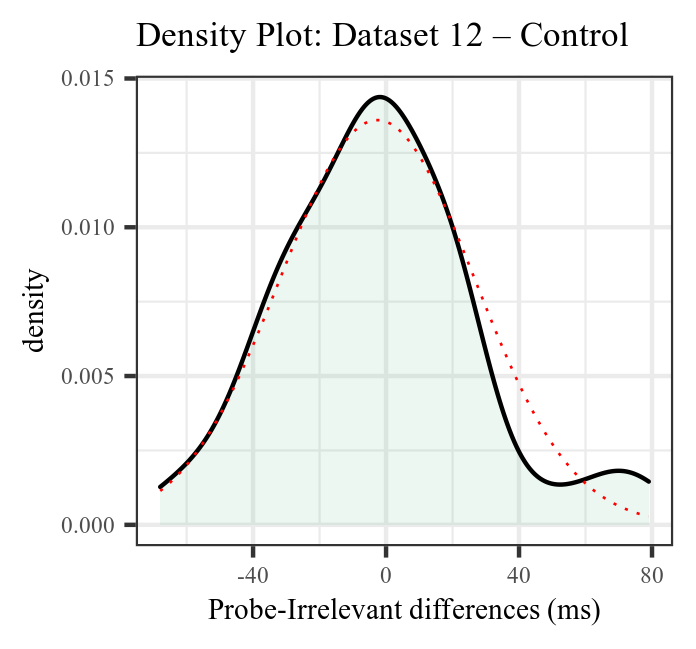

Supplement: S3 File — Figures for the assessment of normal distribution of empirical liar as well as control (truthteller) predictor values (individual probe-irrelevant RT mean differences) in each of the 12 datasets. (ZIP) [file pone.0240259.s009.zip › dataset_12_Control_density_plot.tiff]

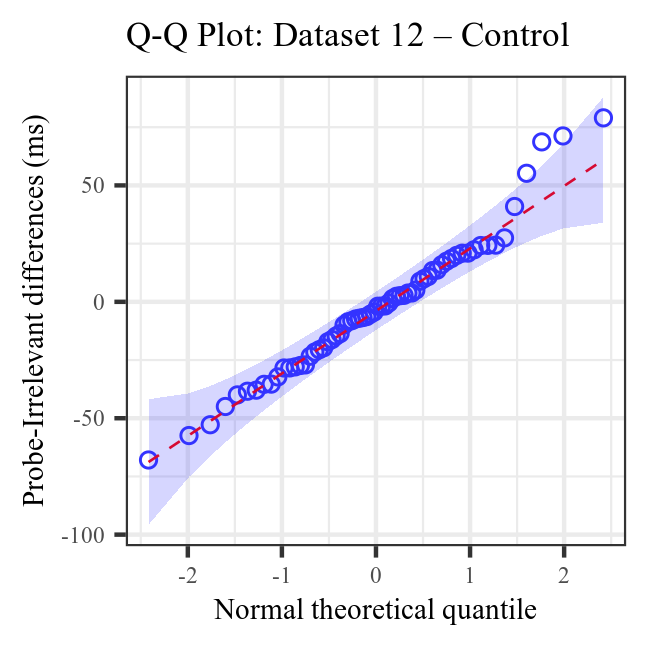

Supplement: S3 File — Figures for the assessment of normal distribution of empirical liar as well as control (truthteller) predictor values (individual probe-irrelevant RT mean differences) in each of the 12 datasets. (ZIP) [file pone.0240259.s009.zip › dataset_12_Control_qq_plot.tiff]

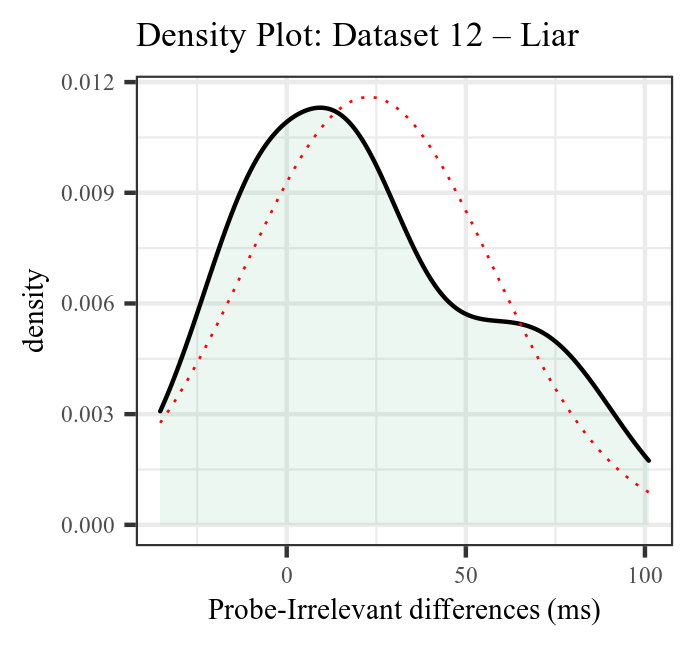

Supplement: S3 File — Figures for the assessment of normal distribution of empirical liar as well as control (truthteller) predictor values (individual probe-irrelevant RT mean differences) in each of the 12 datasets. (ZIP) [file pone.0240259.s009.zip › dataset_12_Liar_density_plot.tiff]

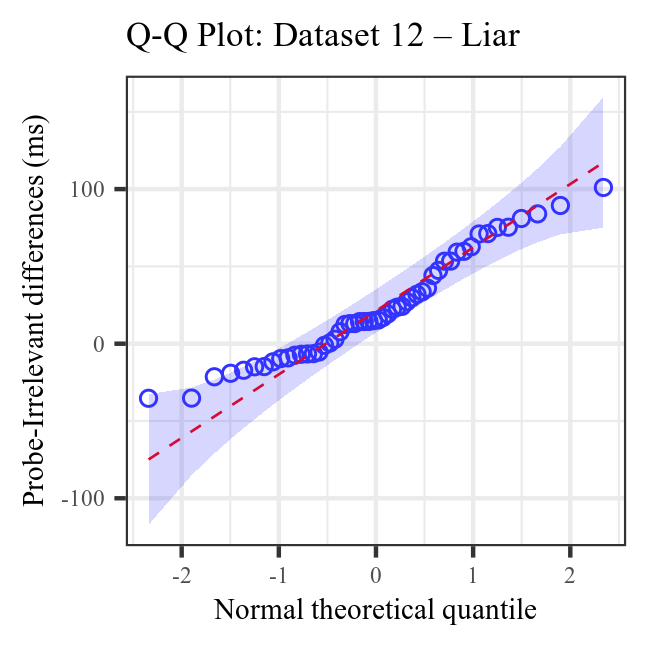

Supplement: S3 File — Figures for the assessment of normal distribution of empirical liar as well as control (truthteller) predictor values (individual probe-irrelevant RT mean differences) in each of the 12 datasets. (ZIP) [file pone.0240259.s009.zip › dataset_12_Liar_qq_plot.tiff]

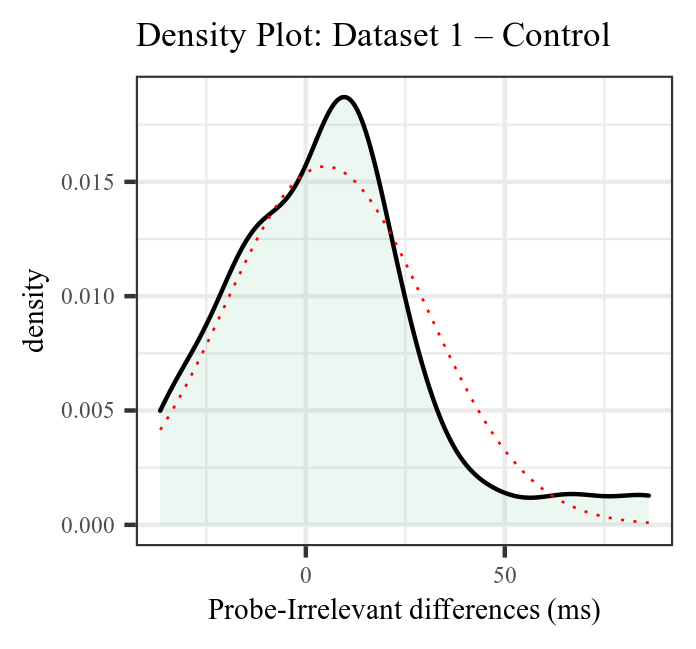

Supplement: S3 File — Figures for the assessment of normal distribution of empirical liar as well as control (truthteller) predictor values (individual probe-irrelevant RT mean differences) in each of the 12 datasets. (ZIP) [file pone.0240259.s009.zip › dataset_1_Control_density_plot.tiff]

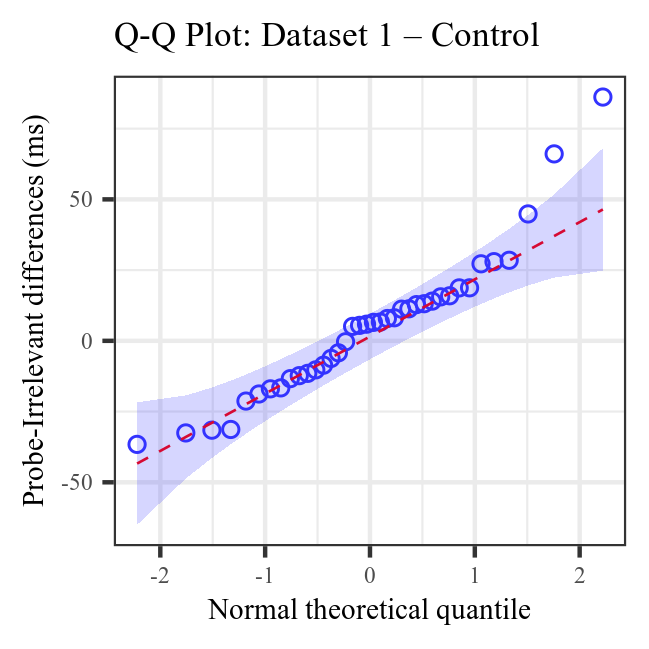

Supplement: S3 File — Figures for the assessment of normal distribution of empirical liar as well as control (truthteller) predictor values (individual probe-irrelevant RT mean differences) in each of the 12 datasets. (ZIP) [file pone.0240259.s009.zip › dataset_1_Control_qq_plot.tiff]

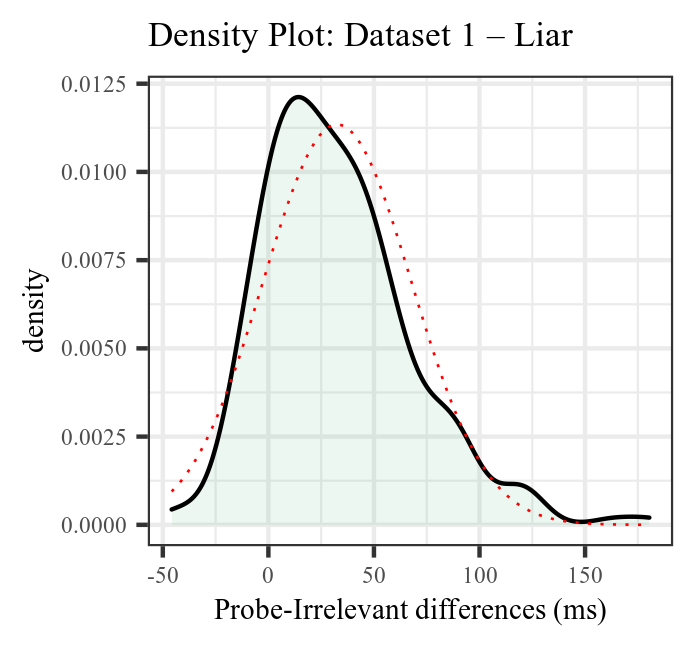

Supplement: S3 File — Figures for the assessment of normal distribution of empirical liar as well as control (truthteller) predictor values (individual probe-irrelevant RT mean differences) in each of the 12 datasets. (ZIP) [file pone.0240259.s009.zip › dataset_1_Liar_density_plot.tiff]

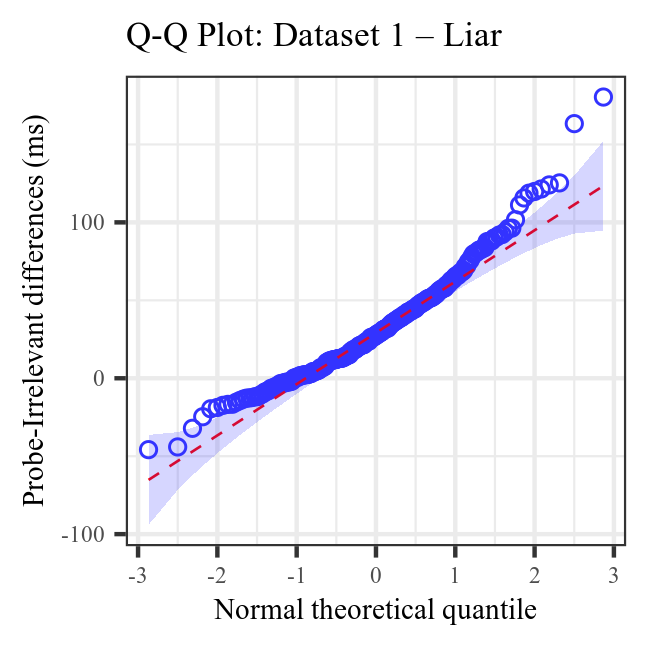

Supplement: S3 File — Figures for the assessment of normal distribution of empirical liar as well as control (truthteller) predictor values (individual probe-irrelevant RT mean differences) in each of the 12 datasets. (ZIP) [file pone.0240259.s009.zip › dataset_1_Liar_qq_plot.tiff]

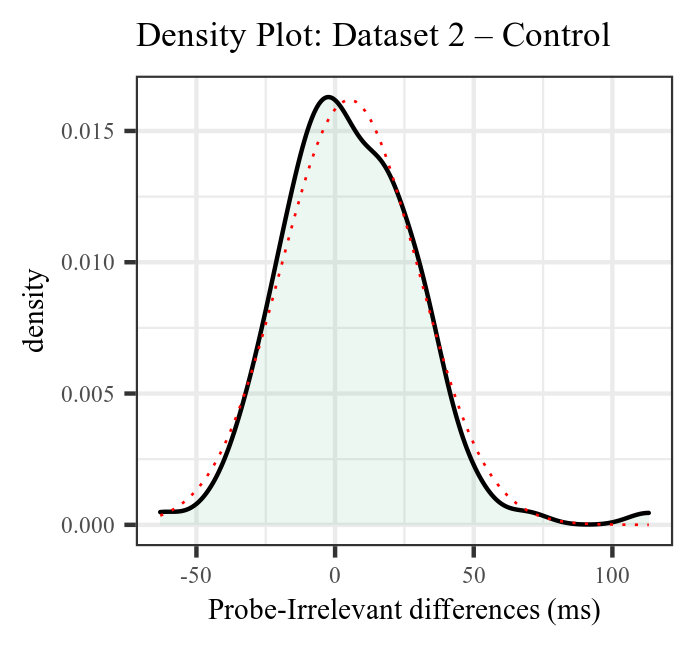

Supplement: S3 File — Figures for the assessment of normal distribution of empirical liar as well as control (truthteller) predictor values (individual probe-irrelevant RT mean differences) in each of the 12 datasets. (ZIP) [file pone.0240259.s009.zip › dataset_2_Control_density_plot.tiff]

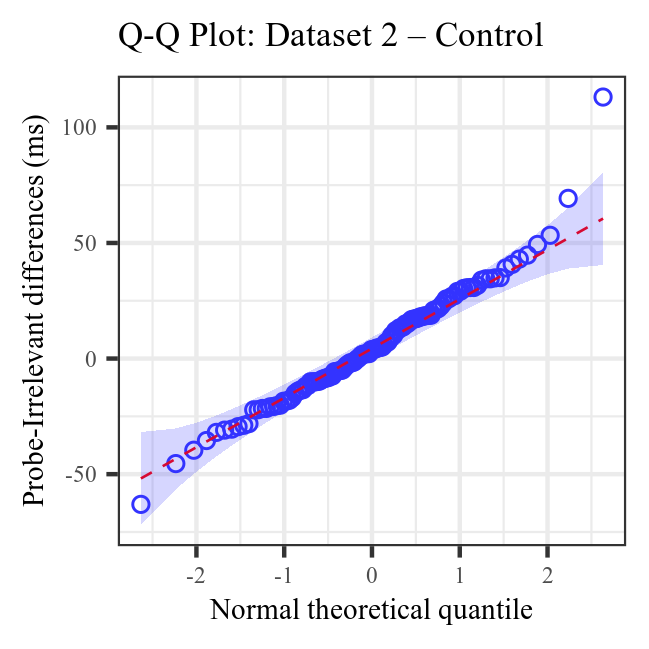

Supplement: S3 File — Figures for the assessment of normal distribution of empirical liar as well as control (truthteller) predictor values (individual probe-irrelevant RT mean differences) in each of the 12 datasets. (ZIP) [file pone.0240259.s009.zip › dataset_2_Control_qq_plot.tiff]

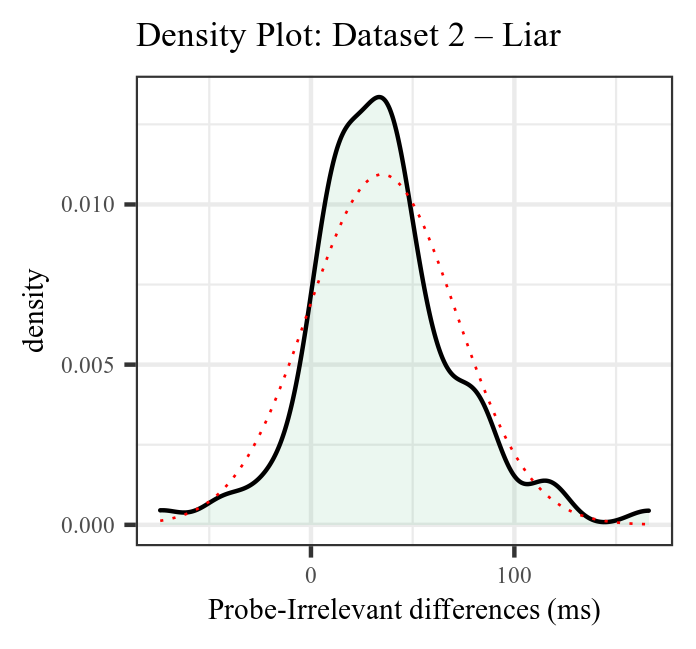

Supplement: S3 File — Figures for the assessment of normal distribution of empirical liar as well as control (truthteller) predictor values (individual probe-irrelevant RT mean differences) in each of the 12 datasets. (ZIP) [file pone.0240259.s009.zip › dataset_2_Liar_density_plot.tiff]

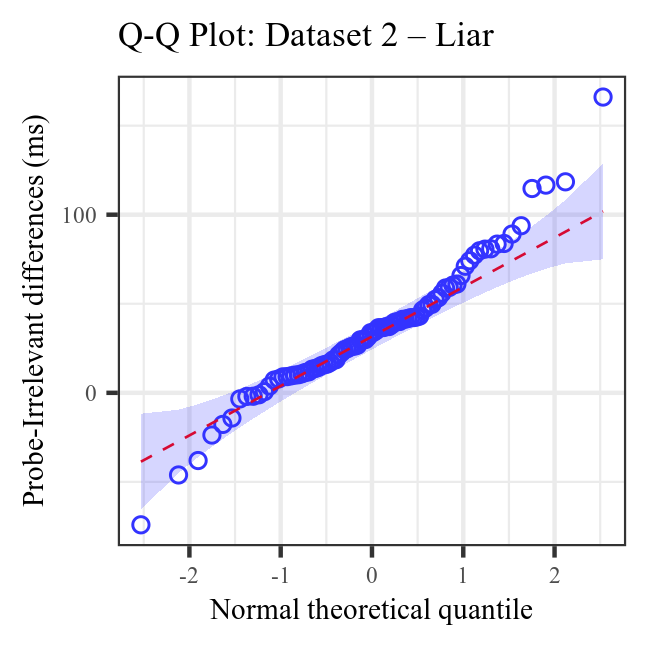

Supplement: S3 File — Figures for the assessment of normal distribution of empirical liar as well as control (truthteller) predictor values (individual probe-irrelevant RT mean differences) in each of the 12 datasets. (ZIP) [file pone.0240259.s009.zip › dataset_2_Liar_qq_plot.tiff]

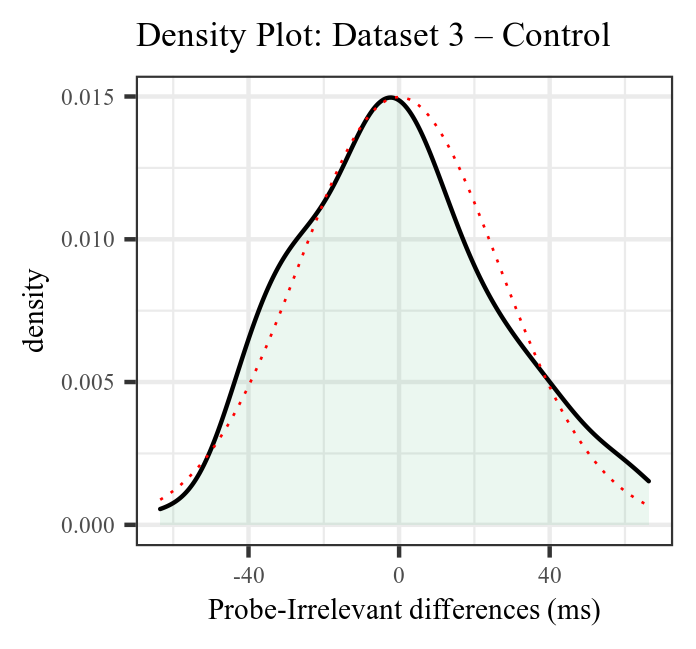

Supplement: S3 File — Figures for the assessment of normal distribution of empirical liar as well as control (truthteller) predictor values (individual probe-irrelevant RT mean differences) in each of the 12 datasets. (ZIP) [file pone.0240259.s009.zip › dataset_3_Control_density_plot.tiff]

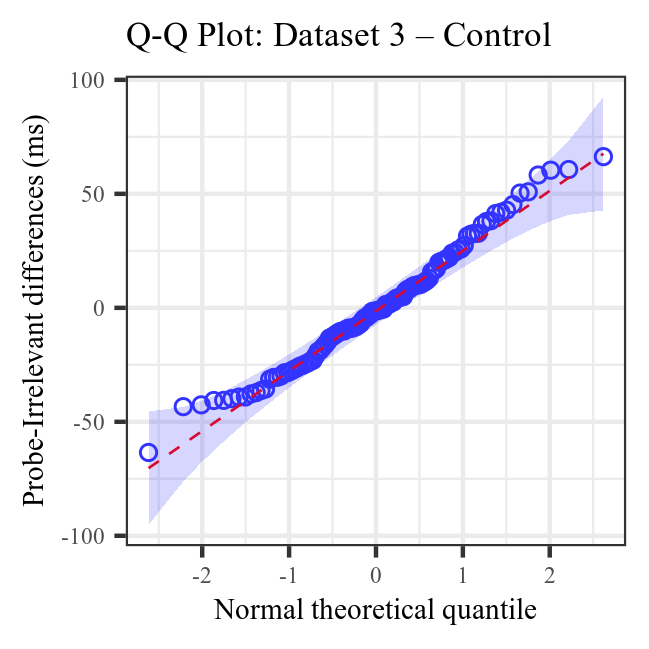

Supplement: S3 File — Figures for the assessment of normal distribution of empirical liar as well as control (truthteller) predictor values (individual probe-irrelevant RT mean differences) in each of the 12 datasets. (ZIP) [file pone.0240259.s009.zip › dataset_3_Control_qq_plot.tiff]

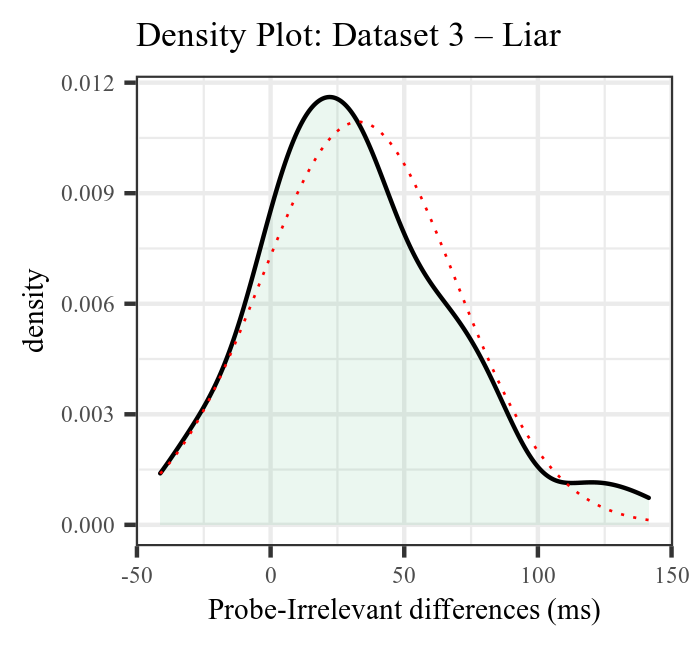

Supplement: S3 File — Figures for the assessment of normal distribution of empirical liar as well as control (truthteller) predictor values (individual probe-irrelevant RT mean differences) in each of the 12 datasets. (ZIP) [file pone.0240259.s009.zip › dataset_3_Liar_density_plot.tiff]

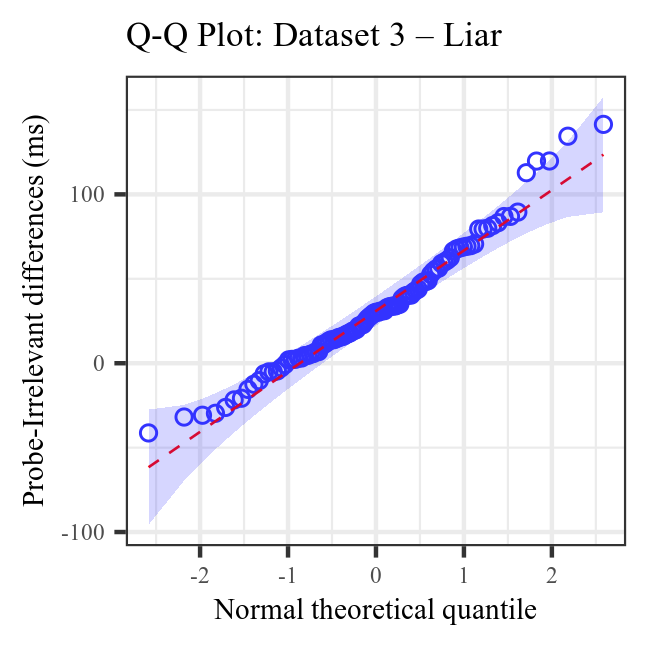

Supplement: S3 File — Figures for the assessment of normal distribution of empirical liar as well as control (truthteller) predictor values (individual probe-irrelevant RT mean differences) in each of the 12 datasets. (ZIP) [file pone.0240259.s009.zip › dataset_3_Liar_qq_plot.tiff]

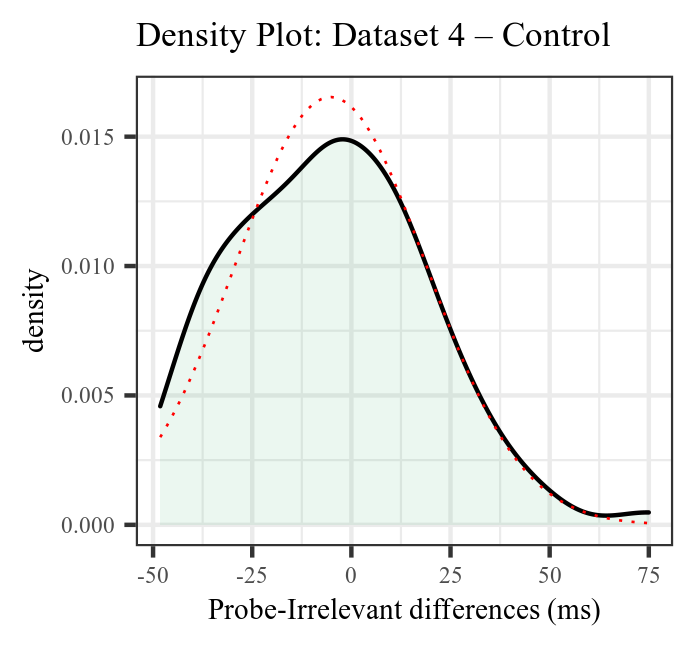

Supplement: S3 File — Figures for the assessment of normal distribution of empirical liar as well as control (truthteller) predictor values (individual probe-irrelevant RT mean differences) in each of the 12 datasets. (ZIP) [file pone.0240259.s009.zip › dataset_4_Control_density_plot.tiff]

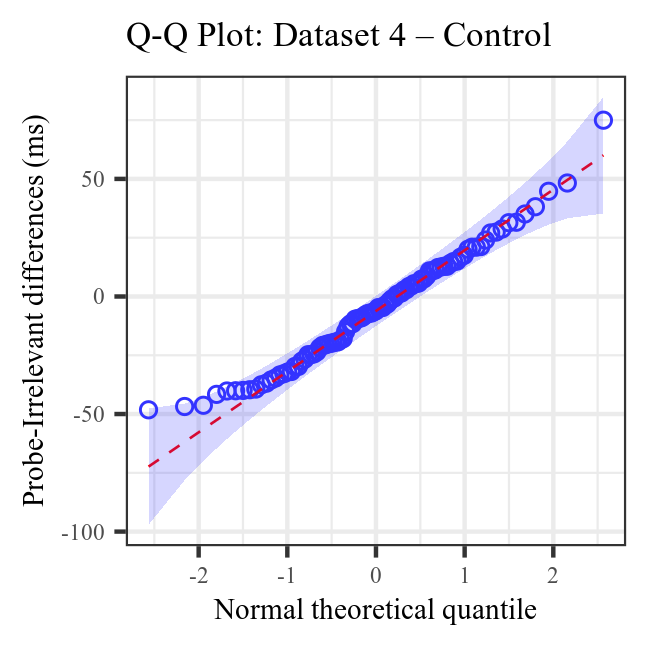

Supplement: S3 File — Figures for the assessment of normal distribution of empirical liar as well as control (truthteller) predictor values (individual probe-irrelevant RT mean differences) in each of the 12 datasets. (ZIP) [file pone.0240259.s009.zip › dataset_4_Control_qq_plot.tiff]

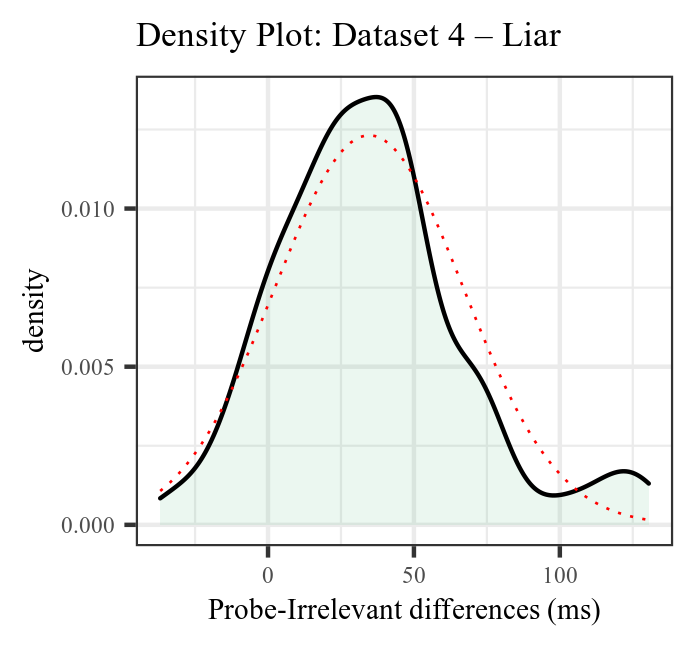

Supplement: S3 File — Figures for the assessment of normal distribution of empirical liar as well as control (truthteller) predictor values (individual probe-irrelevant RT mean differences) in each of the 12 datasets. (ZIP) [file pone.0240259.s009.zip › dataset_4_Liar_density_plot.tiff]

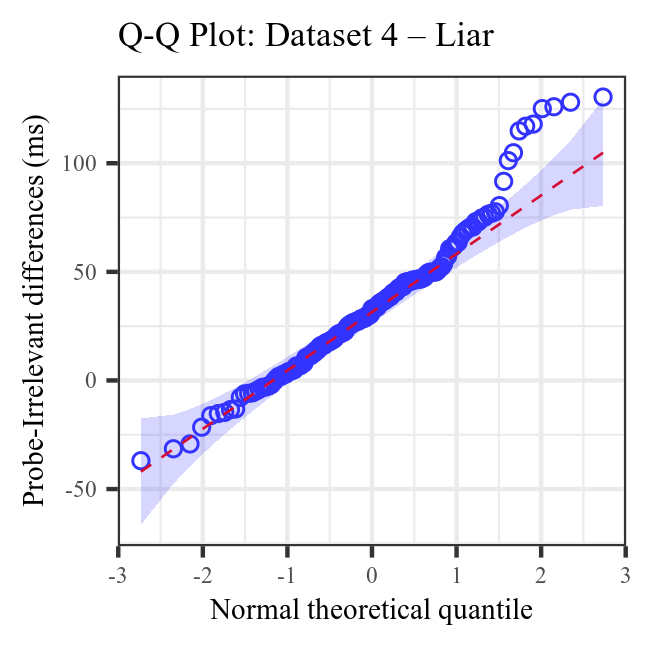

Supplement: S3 File — Figures for the assessment of normal distribution of empirical liar as well as control (truthteller) predictor values (individual probe-irrelevant RT mean differences) in each of the 12 datasets. (ZIP) [file pone.0240259.s009.zip › dataset_4_Liar_qq_plot.tiff]

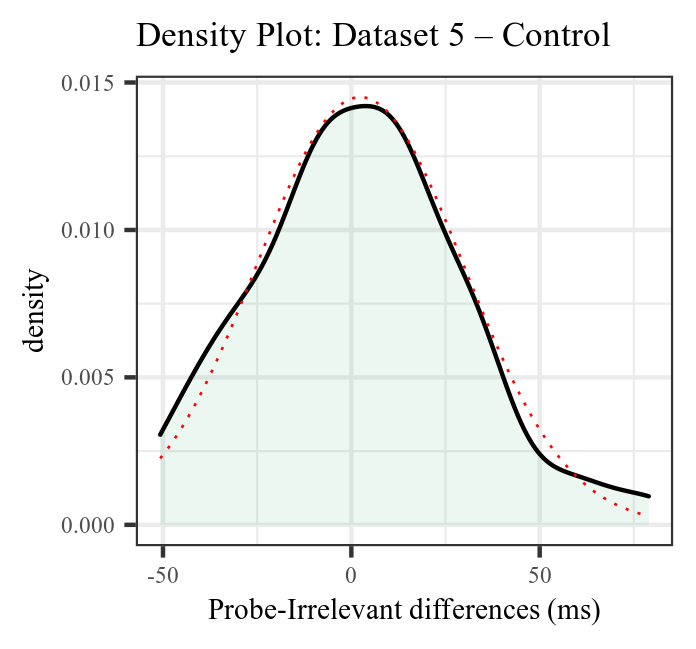

Supplement: S3 File — Figures for the assessment of normal distribution of empirical liar as well as control (truthteller) predictor values (individual probe-irrelevant RT mean differences) in each of the 12 datasets. (ZIP) [file pone.0240259.s009.zip › dataset_5_Control_density_plot.tiff]

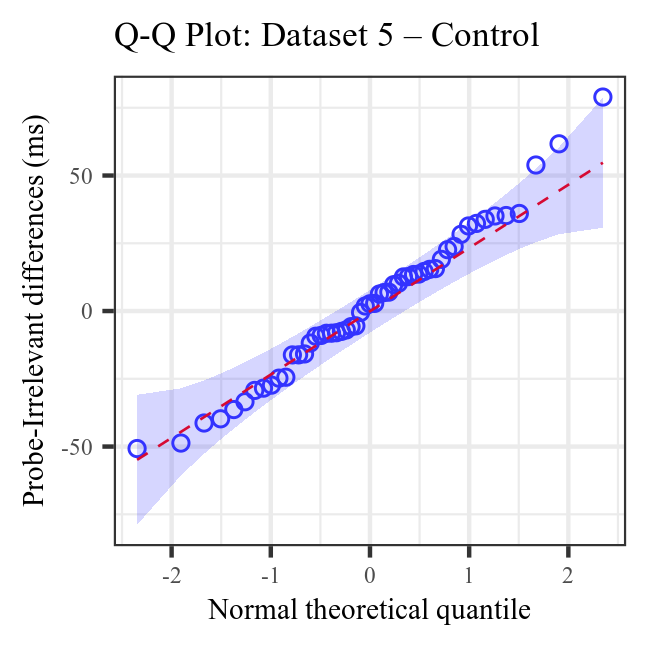

Supplement: S3 File — Figures for the assessment of normal distribution of empirical liar as well as control (truthteller) predictor values (individual probe-irrelevant RT mean differences) in each of the 12 datasets. (ZIP) [file pone.0240259.s009.zip › dataset_5_Control_qq_plot.tiff]

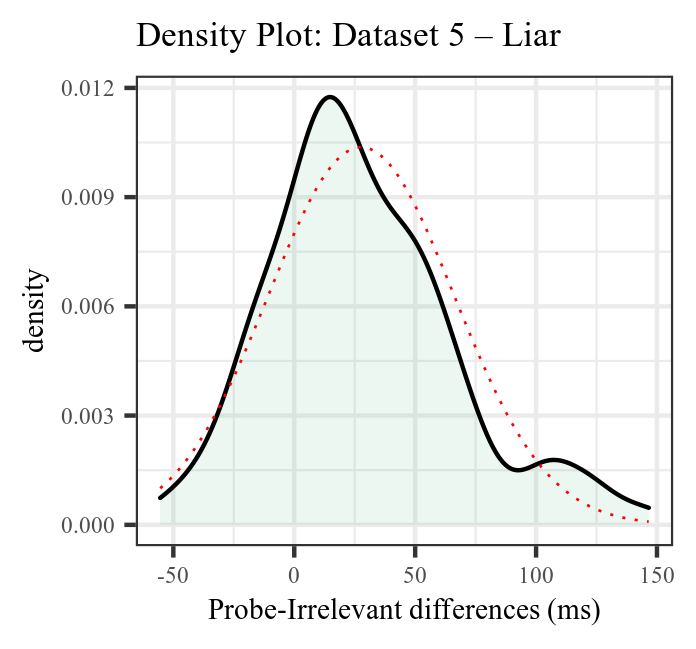

Supplement: S3 File — Figures for the assessment of normal distribution of empirical liar as well as control (truthteller) predictor values (individual probe-irrelevant RT mean differences) in each of the 12 datasets. (ZIP) [file pone.0240259.s009.zip › dataset_5_Liar_density_plot.tiff]

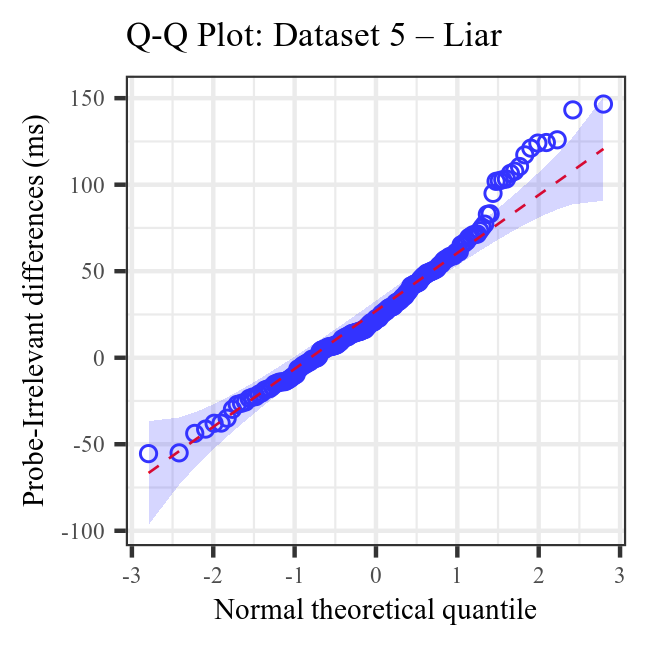

Supplement: S3 File — Figures for the assessment of normal distribution of empirical liar as well as control (truthteller) predictor values (individual probe-irrelevant RT mean differences) in each of the 12 datasets. (ZIP) [file pone.0240259.s009.zip › dataset_5_Liar_qq_plot.tiff]

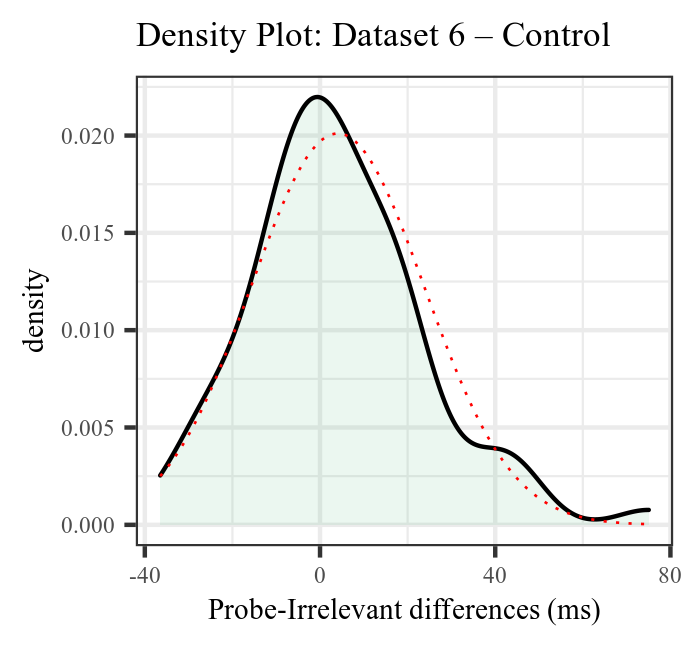

Supplement: S3 File — Figures for the assessment of normal distribution of empirical liar as well as control (truthteller) predictor values (individual probe-irrelevant RT mean differences) in each of the 12 datasets. (ZIP) [file pone.0240259.s009.zip › dataset_6_Control_density_plot.tiff]

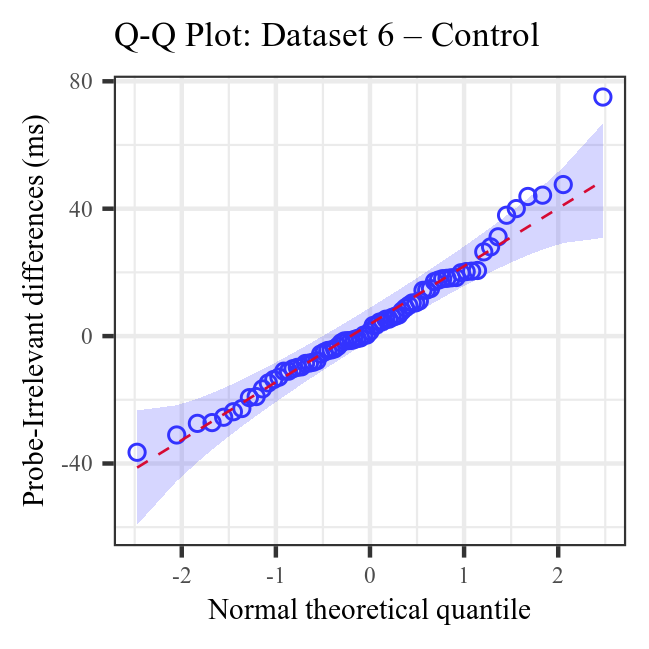

Supplement: S3 File — Figures for the assessment of normal distribution of empirical liar as well as control (truthteller) predictor values (individual probe-irrelevant RT mean differences) in each of the 12 datasets. (ZIP) [file pone.0240259.s009.zip › dataset_6_Control_qq_plot.tiff]

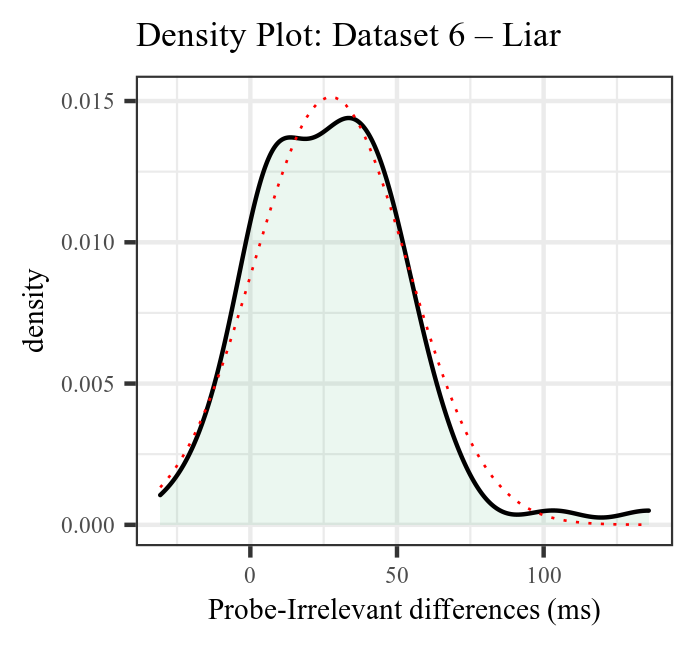

Supplement: S3 File — Figures for the assessment of normal distribution of empirical liar as well as control (truthteller) predictor values (individual probe-irrelevant RT mean differences) in each of the 12 datasets. (ZIP) [file pone.0240259.s009.zip › dataset_6_Liar_density_plot.tiff]

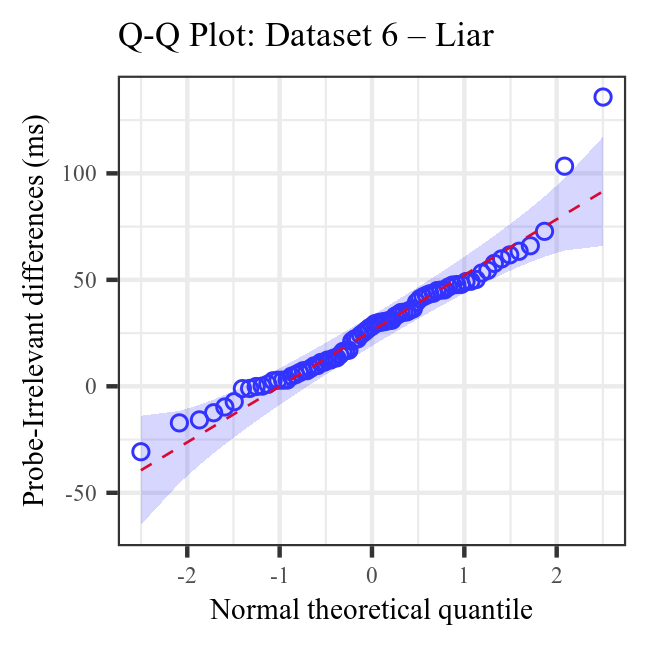

Supplement: S3 File — Figures for the assessment of normal distribution of empirical liar as well as control (truthteller) predictor values (individual probe-irrelevant RT mean differences) in each of the 12 datasets. (ZIP) [file pone.0240259.s009.zip › dataset_6_Liar_qq_plot.tiff]

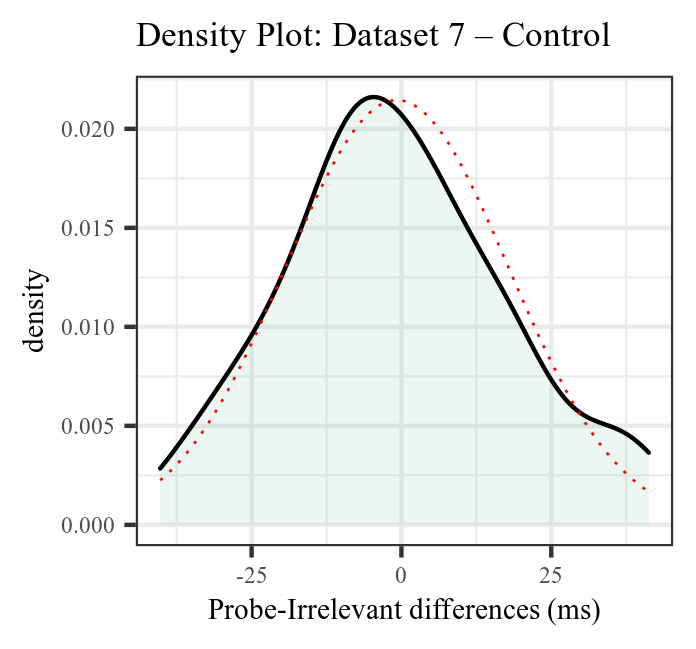

Supplement: S3 File — Figures for the assessment of normal distribution of empirical liar as well as control (truthteller) predictor values (individual probe-irrelevant RT mean differences) in each of the 12 datasets. (ZIP) [file pone.0240259.s009.zip › dataset_7_Control_density_plot.tiff]

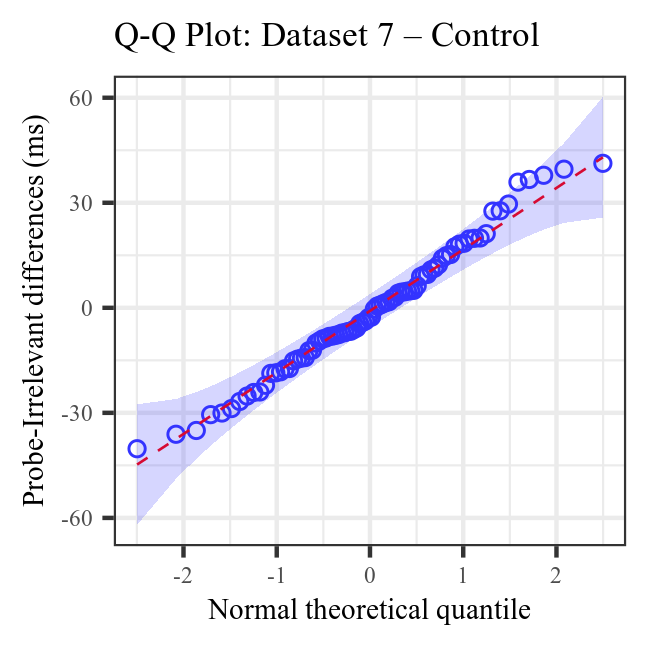

Supplement: S3 File — Figures for the assessment of normal distribution of empirical liar as well as control (truthteller) predictor values (individual probe-irrelevant RT mean differences) in each of the 12 datasets. (ZIP) [file pone.0240259.s009.zip › dataset_7_Control_qq_plot.tiff]

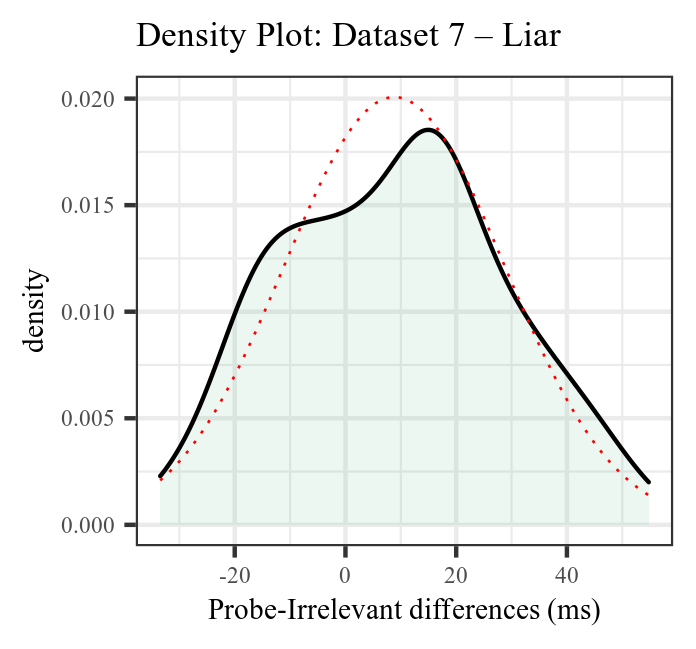

Supplement: S3 File — Figures for the assessment of normal distribution of empirical liar as well as control (truthteller) predictor values (individual probe-irrelevant RT mean differences) in each of the 12 datasets. (ZIP) [file pone.0240259.s009.zip › dataset_7_Liar_density_plot.tiff]

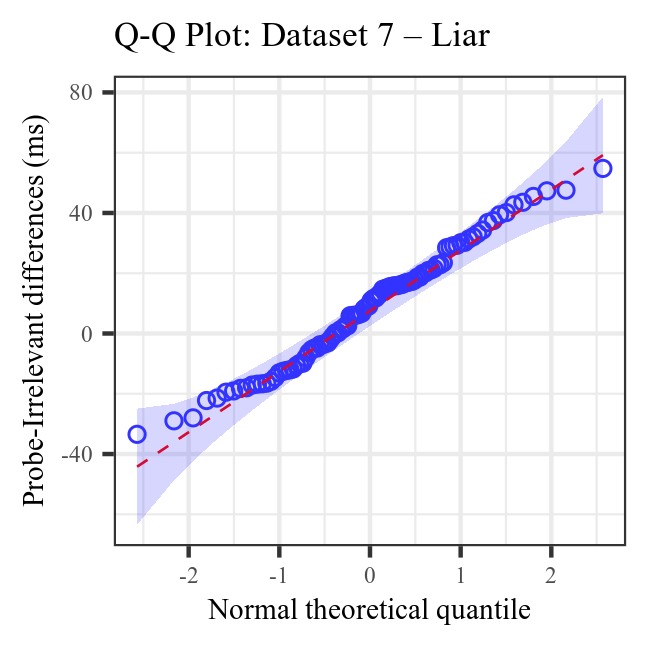

Supplement: S3 File — Figures for the assessment of normal distribution of empirical liar as well as control (truthteller) predictor values (individual probe-irrelevant RT mean differences) in each of the 12 datasets. (ZIP) [file pone.0240259.s009.zip › dataset_7_Liar_qq_plot.tiff]

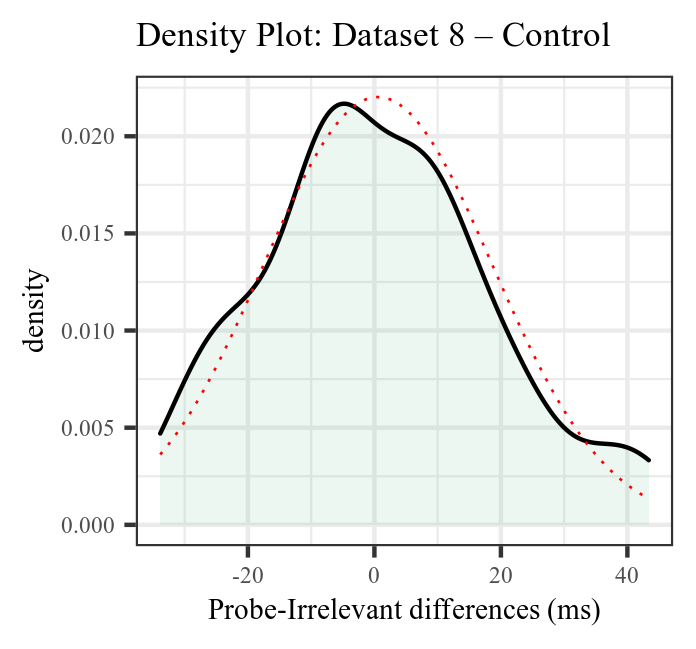

Supplement: S3 File — Figures for the assessment of normal distribution of empirical liar as well as control (truthteller) predictor values (individual probe-irrelevant RT mean differences) in each of the 12 datasets. (ZIP) [file pone.0240259.s009.zip › dataset_8_Control_density_plot.tiff]

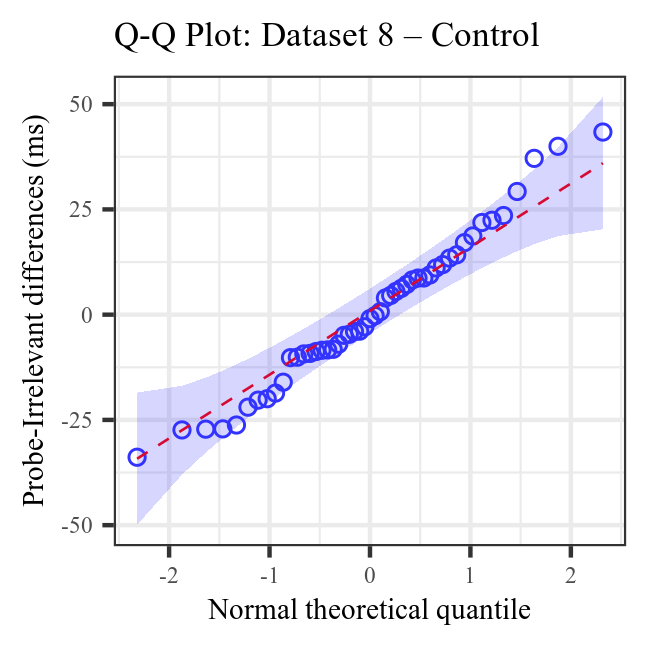

Supplement: S3 File — Figures for the assessment of normal distribution of empirical liar as well as control (truthteller) predictor values (individual probe-irrelevant RT mean differences) in each of the 12 datasets. (ZIP) [file pone.0240259.s009.zip › dataset_8_Control_qq_plot.tiff]

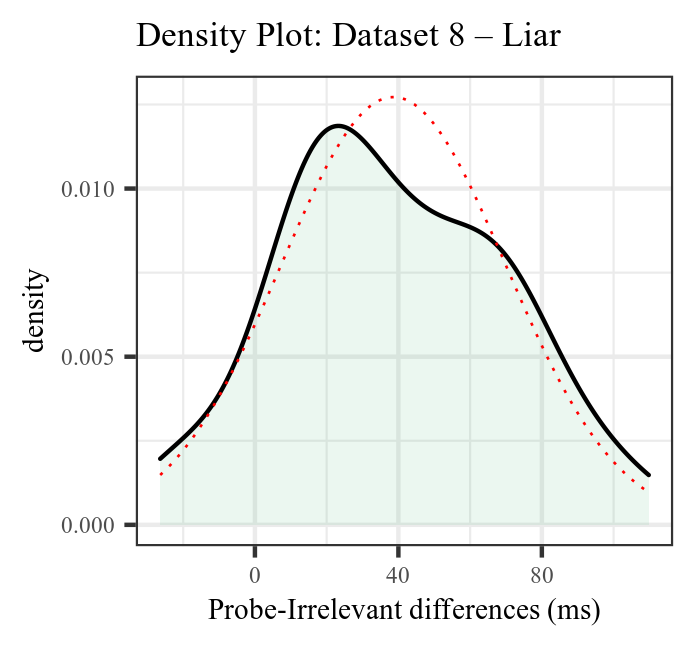

Supplement: S3 File — Figures for the assessment of normal distribution of empirical liar as well as control (truthteller) predictor values (individual probe-irrelevant RT mean differences) in each of the 12 datasets. (ZIP) [file pone.0240259.s009.zip › dataset_8_Liar_density_plot.tiff]

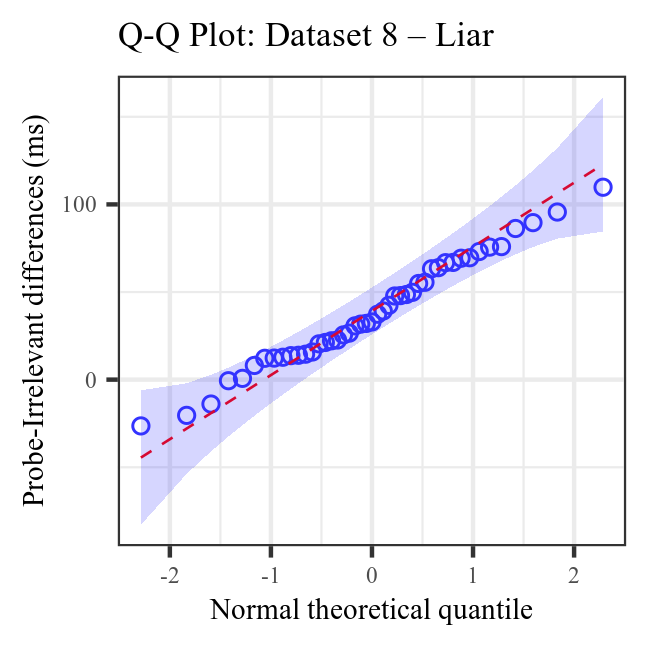

Supplement: S3 File — Figures for the assessment of normal distribution of empirical liar as well as control (truthteller) predictor values (individual probe-irrelevant RT mean differences) in each of the 12 datasets. (ZIP) [file pone.0240259.s009.zip › dataset_8_Liar_qq_plot.tiff]

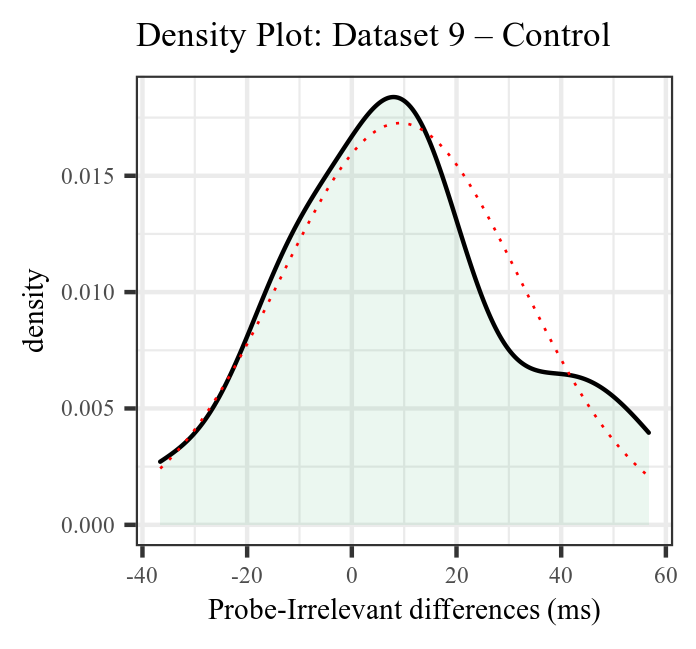

Supplement: S3 File — Figures for the assessment of normal distribution of empirical liar as well as control (truthteller) predictor values (individual probe-irrelevant RT mean differences) in each of the 12 datasets. (ZIP) [file pone.0240259.s009.zip › dataset_9_Control_density_plot.tiff]

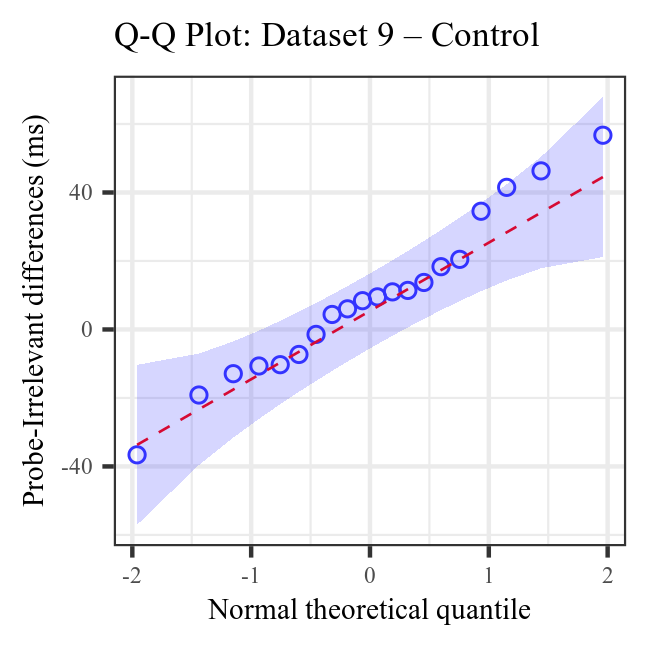

Supplement: S3 File — Figures for the assessment of normal distribution of empirical liar as well as control (truthteller) predictor values (individual probe-irrelevant RT mean differences) in each of the 12 datasets. (ZIP) [file pone.0240259.s009.zip › dataset_9_Control_qq_plot.tiff]

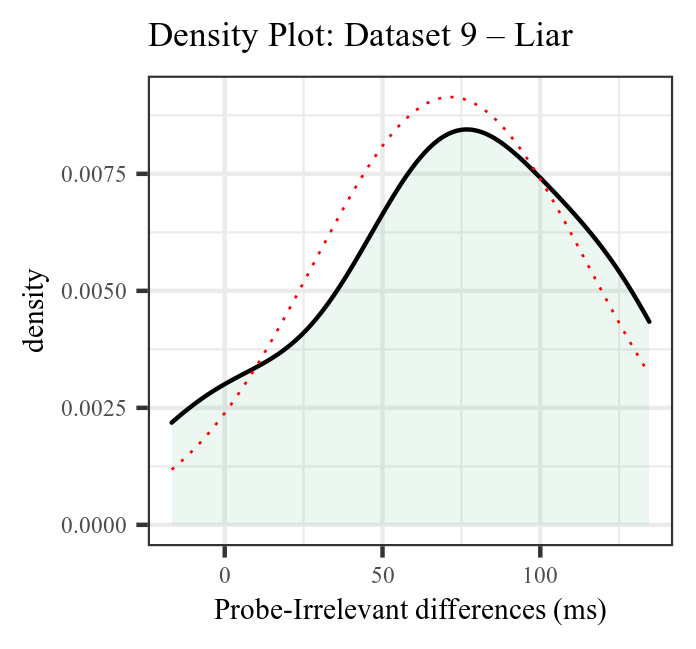

Supplement: S3 File — Figures for the assessment of normal distribution of empirical liar as well as control (truthteller) predictor values (individual probe-irrelevant RT mean differences) in each of the 12 datasets. (ZIP) [file pone.0240259.s009.zip › dataset_9_Liar_density_plot.tiff]

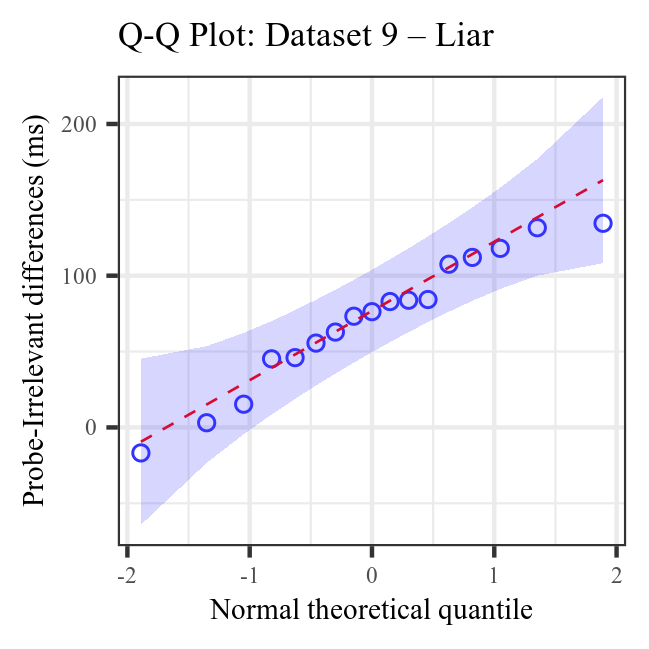

Supplement: S3 File — Figures for the assessment of normal distribution of empirical liar as well as control (truthteller) predictor values (individual probe-irrelevant RT mean differences) in each of the 12 datasets. (ZIP) [file pone.0240259.s009.zip › dataset_9_Liar_qq_plot.tiff]
